# Quantitative Trait Locus Analysis of Mating Behavior and Male Sex Pheromones in *Nasonia* Wasps

**DOI:** 10.1534/g3.116.029074

**Published:** 2016-03-26

**Authors:** Wenwen Diao, Mathilde Mousset, Gavin J. Horsburgh, Cornelis J. Vermeulen, Frank Johannes, Louis van de Zande, Michael G. Ritchie, Thomas Schmitt, Leo W. Beukeboom

**Affiliations:** * Evolutionary Genetics, Groningen Institute for Evolutionary Life Sciences, University of Groningen, Groningen 9700CC, The Netherlands; † Natural Environment Research Council Bio-Molecular Analysis Facility, Department of Animal and Plant Sciences, University of Sheffield, Sheffield S10 2TN, United Kingdom; ‡ Department of Pulmonary Diseases, University Medical Center Groningen, Groningen 9700 RB, The Netherlands; § Population Epigenetics and Epigenomics, Department of Plant Sciences, Technical University Munich, 85354, Freising, Germany; ** Institute for Advanced Study, Technical University Munich, 85748, Garching, Germany; †† School of Biology, University of St. Andrews, St. Andrews KY16 9TH, United Kingdom; ‡‡ Department of Animal Ecology and Tropical Biology, Biocenter, University of Würzburg, Würzburg 97074, Germany

**Keywords:** *Nasonia* courtship, female choice, sex pheromone, QTL analysis, speciation

## Abstract

A major focus in speciation genetics is to identify the chromosomal regions and genes that reduce hybridization and gene flow. We investigated the genetic architecture of mating behavior in the parasitoid wasp species pair *Nasonia giraulti* and *Nasonia oneida* that exhibit strong prezygotic isolation. Behavioral analysis showed that *N. oneida* females had consistently higher latency times, and broke off the mating sequence more often in the mounting stage when confronted with *N. giraulti* males compared with males of their own species. *N. oneida* males produce a lower quantity of the long-range male sex pheromone (4*R*,5*S*)-5-hydroxy-4-decanolide (*RS*-HDL). Crosses between the two species yielded hybrid males with various pheromone quantities, and these males were used in mating trials with females of either species to measure female mate discrimination rates. A quantitative trait locus (QTL) analysis involving 475 recombinant hybrid males (F_2_), 2148 reciprocally backcrossed females (F_3_), and a linkage map of 52 equally spaced neutral single nucleotide polymorphism (SNP) markers plus SNPs in 40 candidate mating behavior genes revealed four QTL for male pheromone amount, depending on partner species. Our results demonstrate that the *RS*-HDL pheromone plays a role in the mating system of *N. giraulti* and *N. oneida*, but also that additional communication cues are involved in mate choice. No QTL were found for female mate discrimination, which points at a polygenic architecture of female choice with strong environmental influences.

Our knowledge of the genetic basis of reproductive isolation is limited (Otte and Endler 1989; Coyne 1992; Coyne and Orr 1998; Macdonald and Goldstein 1999; Presgraves *et al.* 2003; Arbuthnott 2009; Presgraves and Glor 2010; Marie Curie SPECIATION Network 2012; Giesbers *et al.* 2013). Unresolved issues include whether the initial establishment of reproductive isolation is due to a few genes with large effect or many genes with small effect, with strong selection for a few traits or weaker selection on multiple traits, and whether divergence relies on standing genetic variation or new mutations (Rice and Hostert 1993; Barton and Gale 1993; Nosil 2008; Marie Curie SPECIATION Network 2012). With over a million described species and remarkable diversity in form, insects are good models for investigating the mechanisms of reproductive isolation and speciation.

Restrictions to gene flow between species can be divided into factors that act before fertilization, called prezygotic isolation barriers, and those that cause postzygotic isolation after fertilization. Some “speciation genes” causing postzygotic isolation have been identified (Orr 2005; Presgraves and Glor 2010), but less is known about the genetic basis of traits responsible for prezygotic isolation (Arbuthnott 2009). Differences in mating behavior often form primary reproductive barriers, and evolve rapidly through sexual selection in the early stage of the speciation process (Grant and Grant 1997). Indeed, prezygotic isolation seems to evolve at lower levels of overall genetic divergence than postzygotic isolation, at least in sympatry (Coyne and Orr 1989), suggesting that sexual isolation can evolve quickly. Previous studies investigating the genetic architecture of prezygotic isolation barriers in insects have suggested that a polygenic basis is common. For example, in a quantitative trait loci (QTL) study of two closely related cricket species, *Laupala paranigra* and *L. kohalensis*, Shaw *et al.* (2007) found that male calling song differences are due to many genes of small to moderate effect. Gleason and Ritchie (2004) reported six QTL for the differences in male courtship song interpulse interval between *Drosophila simulans* and *D. sechellia*. In a review of the genetic basis of female mate preferences and species isolation in *Drosophila*, Laturney and Moehring (2012) concluded that, although females appear to use the same traits for both within- and between-species mate choice, to some extent a different genetic basis appears to underlie these choices (see also Arbuthnott 2009). In insects, candidate genes for female choice are usually sought among those involved in auditory or olfactory systems or among receptors in the brain that process these signals. Surprisingly, very few studies have considered both male and female mating signals simultaneously, which is required for a full understanding of the genetic basis of prezygotic isolation.

The genus *Nasonia* (Hymenoptera, Pteromalidae) has been used extensively to study the genetics of speciation and species differences (Breeuwer and Werren 1990, 1993, 1995; Gadau *et al.* 1999, 2000, 2002; Bordenstein *et al.* 2000, 2001, 2003; Beukeboom and van den Assem 2001, 2002; Velthuis *et al.* 2005; Niehuis *et al.* 2008, 2010, 2013; Werren and Loehlin 2009; Loehlin *et al.* 2010a, 2010b; Werren *et al.* 2010). *Nasonia* (Hymenoptera: Pteromalidae) are 2–3 mm large parasitoid wasps that sting and lay eggs in pupae of cyclorhaphous flies, such as *Calliphora* and *Protocalliphora*, which are found in bird nests and on carcasses (Whiting 1967; Pultz and Leaf 2003; Grillenberger *et al.* 2008). The *Nasonia* genus contains four closely related species, which diverged 200,000 to 1 million yr ago: *N. vitripennis* (Walker 1836), *N. longicornis*, *N. giraulti* (Darling and Werren 1990), and the recently discovered *N. oneida* (Raychoudhury *et al.* 2010). *N. vitripennis* can be found throughout the world, but the other three species occur only in North America, where their ranges partially overlap. The different species pairs vary in their degree of prezygotic and postzygotic isolation, *N. giraulti* and *N. oneida* exhibit little postzygotic but strong prezygotic isolation (Raychoudhury *et al.* 2010; Giesbers *et al.* 2013).

There are many advantages of *Nasonia* for genetic study of reproductive isolation barriers. One is its haplodiploid reproduction: males are haploid and develop from unfertilized eggs, whereas females are diploid and develop from fertilized eggs. As dominance effects do not exist in haploids, haplodiploidy greatly facilitates quantitative genetic analysis of traits in males, such as genetic linkage mapping and QTL studies (Gadau *et al.* 1999, 2002; Koevoets and Beukeboom 2009; Loehlin *et al.* 2010a, 2010b; Niehuis *et al.* 2011; Gadau *et al.* 2012). Another advantage is the feasibility of interspecific crosses in the laboratory. In nature, the four *Nasonia* species are reproductively isolated due to infection with species-specific strains of *Wolbachia* bacteria that cause cytoplasmic incompatibility and hybrid breakdown in interspecific crosses (Breeuwer and Werren 1990, 1993; Bordenstein and Werren 1998; Bordenstein *et al.* 2001). Antibiotic (*e.g.*, tetracycline) curing in the laboratory allows interspecific crosses and genetic analysis of species-specific traits (Breeuwer and Werren 1990, 1993; Werren and Loehlin 2009; Sharon *et al.* 2011). Other advantages of the *Nasonia* species complex are the availability of full genome sequences of the four species, and high density marker maps (Werren *et al.* 2010). This makes positional cloning of candidate genes identified with QTL studies feasible, as recently demonstrated for a wing size difference (Loehlin *et al.* 2010a, 2010b), and a pheromone dimorphism (Niehuis *et al.* 2013).

In the *Nasonia* species complex, differences in courtship behavior and sex pheromones appear to be responsible for premating isolation between species. All *Nasonia* species perform a complex mating ritual that consists of a series of interactions between the male and female and ends with female receptivity and copulation (Whiting 1967; van den Assem 1986; van den Assem and Werren 1994; Beukeboom and van den Assem 2002; Velthuis *et al.* 2005; Burton-Chellew *et al.* 2007). We previously found 14 QTL for male courtship behavior in interspecific crosses between *N. vitripennis* and *N. longicornis* (J. Gadau, C. Pietsch, J. van den Assem, S. Gerritsma, S. Ferber, L. van de Zande and L. W. Beukeboom, unpublished data). Velthuis *et al.* (2005) reported three major recessive loci for female mate choice between *N. vitripennis* and *N. longicornis*. *Nasonia* males release a long-range sex pheromone to attract virgin females (Ruther *et al.* 2007, 2008), and a different pheromone to induce receptivity in courted females (van den Assem *et al.* 1980; Ruther *et al.* 2010). Niehuis *et al.* (2011) identified genes for alkene biosynthesis with a high similarity to *Drosophila* in a QTL study of cuticular hydrocarbon differences between *N. giraulti* and *N. vitripennis*. Furthermore, Niehuis *et al.* (2013) showed that *N. vitripennis* is the only *Nasonia* species whose males biosynthesize the sex pheromone component (4*R*,5*R*)-5-hydroxy-4-decanolide (*RR*-HDL), besides (4*R*,5*S*)-5-hydroxy-4-decanolide (*RS*-HDL) and 4-methylquinazoline. By genetic mapping and gene knockdown, they narrowed down the genetic basis of enzymes involved in the synthesis of this pheromone component to three closely linked genes on chromosome 1. Finally, following Steiner *et al.* (2006), Buellesbach *et al.* (2013) showed that female cuticular hydrocarbon profiles are used by males as cues for interspecific mate discrimination. However, the genetic basis of female discrimination behavior has not yet been investigated.

In this study, we investigate the species-specific quantitative differences of a component of the male sex pheromone (4*R*,5*S*)-5-hydroxy-4-decanolide and female preference in reciprocal interspecific crosses between *N. giraulti* and *N. oneida*. This is the youngest species pair in the *Nasonia* complex that exhibits strong assortative mating: *N. oneida* females discriminate strongly against *N. giraulti* males, but *N. giraulti* females are less choosy against heterospecific males (Raychoudhury *et al.* 2010). We use QTL analysis with a SNP marker map including candidate genes to investigate the genetic architecture of quantitative changes in male sex pheromone and female preference.

## Material and Methods

### Experimental design and strains

We set up reciprocal interspecific crosses between *N. giraulti* and *N. oneida* using two inbred *Wolbachia*-cured iso-female strains RV2X(u) and NONY11/36TET. RV2X(u) is descended from the wild type *N. giraulti* strain RV2, collected in Rochester, New York (Breeuwer and Werren 1995). NONY11/36TET is derived from *N. oneida* strain NONY11/36, collected in Brewerton, New York (Raychoudhury *et al.* 2010). These are the same strains used for the *Nasonia* Genome Project (Werren *et al.* 2010).

**Figure 1:**
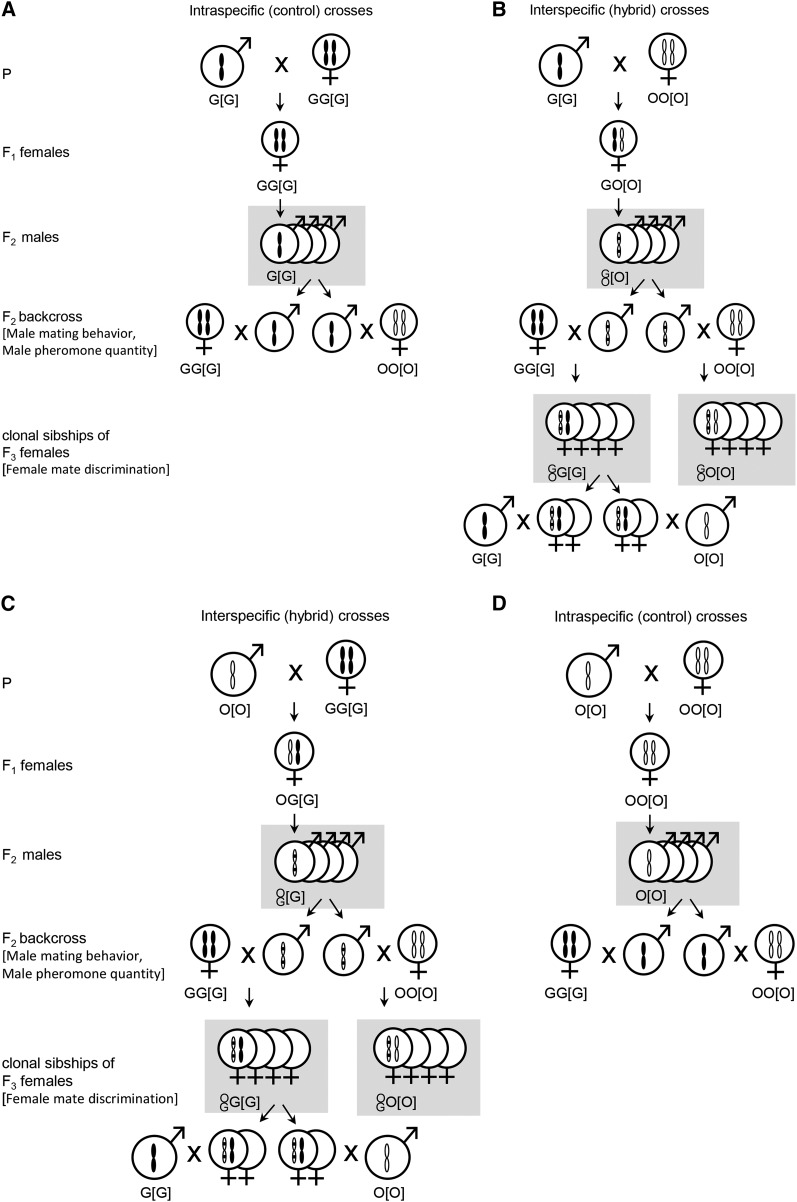
Experimental design. Reciprocal intraspecific and interspecific crosses between *Nasonia giraulti* and *N. oneida*. (A) *N. giraulti* male × *N. giraulti* female (B) *N. giraulti* male × *N. oneida* female (C) *N. oneida* male × *N. giraulti* female and (D) *N. oneida* male × *N. oneida* female. The parental interspecific cross [panels (B) and (C)] generates genetically identical F_1_ hybrid females that produce unique recombinant F_2_ hybrid male offspring of a 50:50 genetic mixture of the two parental species. Backcrossing of F_2_ hybrid males to parental strain females yielded sibships of hybrid F_3_ females with a 75:25 ratio of either parental species’ genome. The haploid hybrid male genotype is indicated with the paternal species on top and the maternal species below. The diploid hybrid F_3_ backcrossed female genotype consists of a recombined hybrid set, and a pure species set, resulting in 75% genome of one species, and 25% of the other species on average. The diploid hybrid female genotype is indicated with the paternal species first followed by the maternal species. The square brackets indicate species cytoplasm, which is maternally inherited. Male mating behavior and male pheromone quantity were investigated of individual F_2_ hybrid males (gray shading), and female mate discrimination was investigated on clonal sibships of F_3_ females (gray shading). G, *N. giraulti*; O, *N. oneida*.

Wasps were cultured in a climate room with constant temperature at 25°, 16:8 h light/dark cycle, and 45% relative humidity. Reciprocal interspecific crosses were set up by mating males of one species with virgin females of the other species. F_1_ virgin females were collected and allowed to oviposit to generate recombinant F_2_ hybrid haploid males. These males were subsequently mated to females of either of the two pure species to generate a F_3_ generation that is referred to as clonal sibships of hybrid F_3_ females (Figure 1). Individuals within a sibship have identical genotypes, as their hybrid fathers are recombinant haploids and produce identical sperm, whereas their mothers are derived from pure species lines that are iso-female and inbred. This experimental set-up allowed for repeated testing of identical genotypes and a quantitative, rather than binary, estimate of mate discrimination (see below).

### Behavioral assays

The typical courtship behavior of *Nasonia* includes several stages (van den Assem 1986; van den Assem and Beukeboom 2004; Clark *et al.* 2010). The male produces a long-range sex pheromone as described above to attract the female. After a latency period, the male recognizes the cuticular hydrocarbon profile of the female and mounts the female (Buellesbach *et al.* 2013). Subsequently, he starts his display by touching the antennae of the female with its own, followed by movements of the head, called “head-nods,” together with vibration of the wings, followed with a pause. This pattern of head-nods and pauses is termed a cycle, which is repeated several times. At each first head nod in a cycle, the male deposits an as yet unidentified short-range pheromone (aphrodisiac) on the female’s antennae. After a number of cycles, the female will either accept the male by lowering her antennae and raising her abdomen for copulation, or reject the male, who will dismount and terminate courtship. Successfully mated males back up on top of the female after copulation to perform postcopulatory courtship consisting of a few similar cycles of head-nods and pauses with a pheromone released, and subsequently dismount.

Observations were performed in a climate room with constant temperature at 25°, a 16:8 h light/dark cycle and 45% relative humidity. Virgin males and virgin females were placed individually in glass tubes (height 30 mm, diameter 10 mm) 1 d prior to the mating trial to allow plenty time for males to release the long-range sex pheromone, as evidenced by white dots marked by males on the surface of the glass tubes (Steiner and Ruther 2009). Males and females were subsequently paired in no-choice experiments by joining the two glass tubes. Couples were then observed under a stereo binocular microscope until first copulation, or for a maximum of 10 min. All females and males were used only once.

The following sequential courtship components were scored: (a) “interest”, males and females approached each other; (b) “cross direction”, which sex passed the border of the two joint glass tubes first; (c) “border cross time”, the time when the first wasp passed the border of the two joint glass tubes; (d) “latency time”, the time from the start of the tube joining until the male mounting on the female; (e) “mounting”, the male mounted on top of the female; (f) “arrest”, the female becomes immobile after the male’s mounting; (g) “display”, the male performs repeated cycles of head-nods and wing vibrations interrupted with pauses; (h) “attempted copulation”, the male attempts to copulate; (i) “normal copulation”, copulation occurs. After observation, males were frozen at –20° for chemical and DNA analysis, and females were given two fly pupae as host to produce offspring. For each reciprocal cross, about 250 individuals were divided in two groups of 125 males to be crossed to either parental species females, resulting in the observation of 494 48- to 72-hr-old F_2_ hybrid males. In the next generation, female mate discrimination was measured with the same experimental set-up. A total of 2922 24- to 48-hr-old virgin hybrid F_3_ females were tested with a virgin pure male of either parental strain. Female mate discrimination was scored as “mate rejection”, *i.e.*, when the male mounted the female but the female did not become receptive (Velthuis *et al.* 2005). In most cases five females were tested per sibship, and the mate discrimination values could take a value between 0 (all females within a sibship accepted their mate) and 1 (all females rejected their mate). Pairs in which the male did not mount the female within the 10 min of observation were discarded, resulting in a total of 2210 hybrid F_3_ females, of which 2148 (578 sibships) with genotypic information were used for QTL mapping of mate discrimination.

### Chemical analysis

*N. giraulti* males produce a sex pheromone composed of (4*R*,5*S*)-5-hydroxy-4-decanolide (*RS*-HDL) and 4-methylquinazoline in an abdominal gland in large amounts to attract females (Niehuis *et al.* 2013; Ruther *et al.* 2014). To quantify the amount of *RS*-HDL, males were killed after behavioral assays by freezing them at –20°. Pheromones were extracted by immersing the male abdomen of each F_2_ individual in 80 μl CH_2_CL_2_ [containing 88 ng methyl palmitate (1.1 ng/μl) as an internal standard] in glass vials for 2 hr. After pheromone extraction, the abdomen was removed from the solvent, and extracts were stored at –20°. Next, the solvent of the extracts was evaporated up to ∼1 μl using a gentle stream of nitrogen. The entire residual volume was used for the chemical analysis. Gas chromatography/mass spectrometry (GC/MS) analysis was performed with a HP 6890 gas chromatograph coupled with a HP 5973 Mass Selective Detector (Hewlett Packard, Waldbronn, Germany). The GC (split/splitless-injector in splitless mode for 1 min, injected volume: 1 µl at 250°) was equipped with a DB-5 Fused Silica capillary column (30 m × 0.25 mm ID, df = 0.25 μm, J&W Scientific, Folsom, CA). Helium served as a carrier gas with a constant flow of 1 ml/min. The following temperature program was used: start temperature 60°, temperature increase by 5° per minute up to 300°, and kept at 300° for 10 min. The electron ionization mass spectra (EI-MS) were acquired at an ionization voltage of 70 eV (source temperature: 230°). The software HP Enhanced ChemStation G1701AA Version A.03.00 was used for recording and analysis of chromatograms and mass spectra. Quantification via integrated peak areas and internal standard was performed using the same software.

### SNP markers development

Using the *Nasonia* genome sequences, a total of 57 single nucleotide polymorphisms (SNP) markers were developed with 10 cM intervals covering all five chromosomes. The available *N. giraulti* .bam file (Werren *et al.* 2010), and *N. oneida* short reads FASTA file (Human Genome Sequencing Center at Baylor College of Medicine), were assembled on *N. vitripennis* scaffolds Nvit_1.0 version. In order to estimate the position of the SNPs on the genetic map, the aligned sequences of *N. giraulti* and *N. oneida* were compared using CLC Genomics Workbench (CLC bio A/S) to those containing the SNP markers between *N. giraulti* and *N. vitripennis* developed by Niehuis *et al.* (2010). Following the same procedure, an additional 50 SNPs were identified in candidate genes that are known to be associated with mating behavior, courtship song, circadian rhythm, and sex pheromones (Supplemental Material, Table S1). Identification of candidate genes used information from *Nasonia* and *Drosophila* courtship behavior, courtship song, circadian rhythm, pheromone production, and pheromone detection (Gleason *et al.* 2005; Kankare *et al.* 2010; Niehuis *et al.* 2013).

For confirmation, all developed SNPs on chromosome 1 were checked for their amplification and polymorphism in *N. giraulti* and *N. oneida*. After primer design with Primer3, DNA was amplified by PCR with annealing temperature of 58°, and Sanger sequenced on an ABI3130xl or ABI3730. Sequences were checked and validated with Chromas (version 2.33, Technelysium Pty Ltd), and aligned with MEGA5 (Tamura *et al.* 2011). Sequences were blasted to the *N. giraulti* and *N. oneida* scaffolds with CLC Genomics Workbench to verify the position of the SNP. After these initial confirmatory tests, chip design was performed by Illumina (San Diego, CA). Only the SNPs with quality scores of at least 0.6 were placed on the chip, consisting of 55 background markers and 41 candidate genes.

### SNP genotyping

DNA was extracted from the frozen heads and thoraxes of individual F_2_ hybrid males, and the two parental couples (whole wasps) with a standard high salt chloroform protocol (Maniatis *et al.* 1982). SNP genotyping of 480 DNA samples was performed in five 96-semi-skirt-plates, including 476 F_2_ hybrid males and four parental wasps, using the GoldenGate Genotyping assay on the Illumina BeadExpress platform of NERC Bio-molecular Analysis Facility (Project NBAF653) at University of Sheffield, UK. The BeadExpress raw data were processed using Illumina GenomeStudio (version 2011.1) to determine the sample genotypes, based on signal intensity (“R”) and allele frequency (the ratio of signal intensities for the two possible bases, “Theta”), relative to the cluster position for a given SNP marker.

The most crucial aspect of SNP analysis is to define boundaries between clusters of genotypes. A cluster is a group of SNPs that fall into either one of the homozygous alleles or the heterozygous alleles (Hoffman *et al.* 2012). *Nasonia* males, being haploid, provide a great advantage in this respect because they have only two homozygote (hemizygote) classes and therefore typically have clear clusters. Any SNP with only one cluster or low call frequency in a cluster was discarded (four out of 96 SNPs, three background markers and one in a candidate gene). In total, genotype information of 475 hybrid males with 92 well-typed SNP markers was successfully obtained for further QTL analysis (Table S1).

### Statistical analysis

Statistical analysis of all phenotypic data was performed with the R statistical software (version 3.1.2). A generalized linear model (glm) was used to identify the effect of different factors in the F_2_ hybrid male crosses, including male genotype, female partner species, their interaction, and cross type, on all investigated behavioral traits and pheromone quantity. The glm for F_3_ females included female genotype, male partner species, their interaction, and cross type on female mate discrimination. The best statistical model was built up from a full model with all possible factors, followed by removal of nonsignificant explanatory factors. Chi-square (χ^2^) tests were used to compare the likelihood of the different models. *Post hoc* tests (*e.g.*, Tukey or multiple comparisons) were subsequently performed to confirm the differences between groups. ANOVA was used to estimate the effect of partner, mother, father, grandmother, and grandfather species and their interactions, on all the investigated traits in hybrid crosses. ANOVA was also used to estimate the between- and within-sibship variance in hybrid female mate discrimination as a proxy for broad-sense heritability of this trait. The between-sibship variance is the total phenotypic variance (*V*_P_) and the within-sibship variance is the environmental variance (*V*_E_) as all genotypes within a sibship are identical.

### Genetic mapping and QTL analysis

The R/QTL package in R (version 3.1.2) was used to identify genetic regions contributing to the variation in phenotypic quantitative traits (Broman and Sen 2009). A linkage map was generated from the genotypes of 475 F_2_ hybrid males with the set of 92 SNP markers. F_3_ female genotypes were inferred from the genotypes of their F_2_ recombinant father and their pure species mother (iso-female line). Recombination fractions between all pairs of markers were estimated using the Lander-Green algorithm (Lander and Green 1987) to obtain precise genetic distances between markers of different linkage groups. A likelihood of odds (LOD) score was calculated for each individual at each marker according to Lincoln and Lander (1992) to correct for genotyping errors in map construction. The map did not deviate from the expected marker order and spacing based on the annotated genome.

R/QTL uses a hidden Markov model to calculate QTL genotype probabilities and effects given the observed marker data, with allowance for genotyping errors. All QTL scans of F_2_ male traits were performed separately for both backcross types. QTL scans of F_3_ female mate discrimination were performed separately for either partner species. First, standard interval mapping was applied (using the “scanone” function) with a single-QTL genome scan with a stepsize of 1 cM, with a normal model for all phenotypic traits based on Haley-Knott regression (Haley and Knott 1992). Permutation tests based on 10,000 permutations yielded 5% genome-wide LOD significance thresholds. Next, a two-dimensional genome scan with a stepsize of 2 cM with Haley-Knott regression was performed (using the “scantwo” function), allowing the estimation of additive and epistatic effects by evaluating several types of models, (1) a full model of additive and epistatic effects, (2) an additive model, (3) an epistatic model, (4) a comparison between the full model and the best single-QTL model, and (5) a comparison between the additive model and the best single-QTL model. Finally, a multiple-QTL model was fit with QTL identified from the one- and two-QTL scan (using function “fitqtl”). The fit of the two-QTL model was compared to the reduced model in which a single-QTL is omitted. Possible interactions between the detected QTL and other potential QTL in the multiple-QTL model were investigated (using the “addint” and “addqtl” functions respectively). Next, a fully automated stepwise algorithm (function “stepwiseqtl”) optimized the penalized LOD scores (Manichaikul *et al.* 2009), which yielded the best final QTL model. Separate QTL analysis for partner species yielded partner-specific QTL for some of the traits, suggesting QTL × partner interactions. To quantify this interaction effect, we performed additional QTL scans, which included partner as a covariate and the QTL × partner as an interaction covariate.

Wasp strains are available upon request. File S1 presents ID numbers and genomic locations of SNPs of Table S1. File S2, File S3, and File S4 present phenotypic data of courtship behavior, male pheromone quantity, and female mate discrimination. File S5 contains genotypic data of recombinant F_2_ hybrid males.

### Data availability

The authors state that all data necessary for confirming the conclusions presented in the article are represented fully within the article.

## Results

### Phenotypic analysis

#### Courtship behavior in pure species and hybrid males:

Females were the first to walk over to the male in 75–88% of the cases, and this did not differ between *N. giraulti* and *N. oneida* in pure and hybrid crosses (glm, effect of females: χ^2^ = 1.20, *P* = 0.274; effect of males: χ^2^ = 5.90, *P* = 0.116; no interaction effect and no cross type effect). Border cross times also did not differ between species in pure and hybrid crosses (glm, effect of females: χ^2^ = 0.63, *P* = 0.427; effect of males: χ^2^ = 6.06, *P* = 0.109; no interaction effect and no cross type effect).

**Figure 2:**
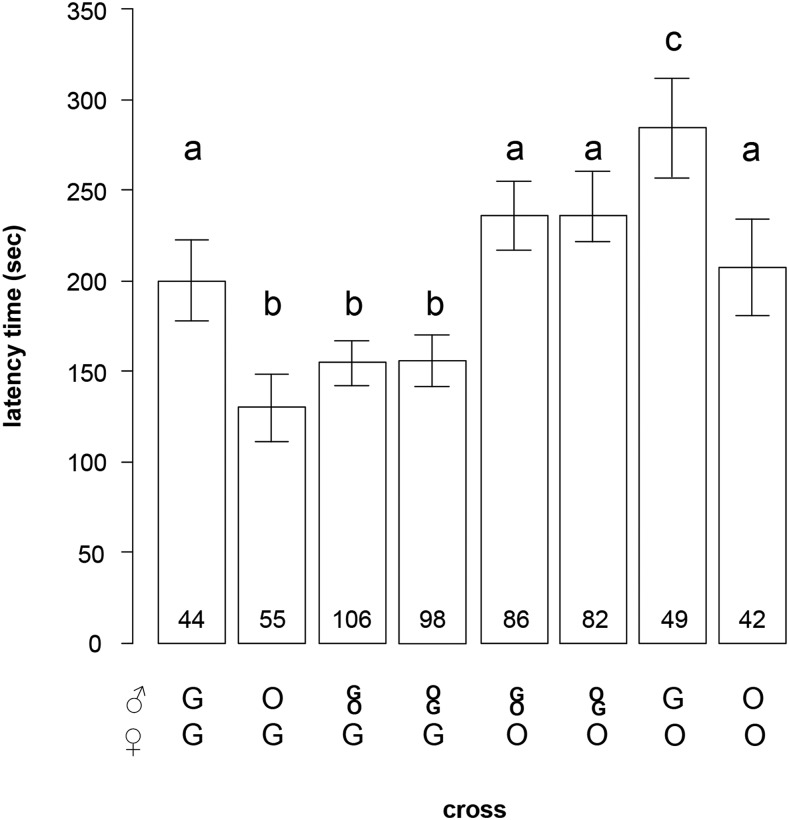
Latency time (mean ± SE) of pure species and hybrid males crossed with either parental species females. Crosses involving *N. oneida* females show significantly longer latency times. The genotype labeling is as in Figure 1. Sample sizes are shown within the bars. Different lower case letters indicate significant differences in means between crosses (glm, Tukey test, *P* < 0.05).

Latency times did not differ between the pure species crosses, but differed significantly in interspecific crosses and crosses with hybrid males (Figure 2). *N. oneida* females always had longer latency times than *N. giraulti* females when they have the same male partner species (glm, effect of females: χ^2^ = 38.22, *P* < 0.001; effect of males including the hybrids: χ^2^ = 12.22, *P* = 0.007; no significant interaction effect; cross type effect: χ^2^ = 48.82, *P* < 0.001). Surprisingly, latency times of *N. giraulti* females are shorter in crosses with *N. oneida* males than in crosses with their own species males (mean ± SE; 129.7 ± 18.3 sec *vs.* 199.9 ± 22.6 sec, *t*-test, *t*_87.83_ = 2.42, *P* = 0.018). Latency times of *N. oneida* females are longer in interspecific than intraspecific crosses (mean ± SE; 284.8 ± 27.2 sec *vs.* 207.5 ± 26.5 sec, *t*-test, *t*_88.76_ = 2.04, *P* = 0.045). In crosses with hybrid males, latency times are also longer for *N. oneida* females than *N. giraulti* females (mean ± SE; 238.4 ± 13.6 sec *vs.* 155.8 ± 9.7 sec, *t*-test, *t*_312.15_ = 4.99, *P* < 0.001) albeit with a strong male partner species effect (Table S3, ANOVA, *F*_1,367_ = 25.56, *P* < 0.001). These results indicate that the longer latency times of the interspecific crosses are reduced in the hybrid male crosses, consistent with the intermediate genotype of the hybrid males.

**Figure 3:**
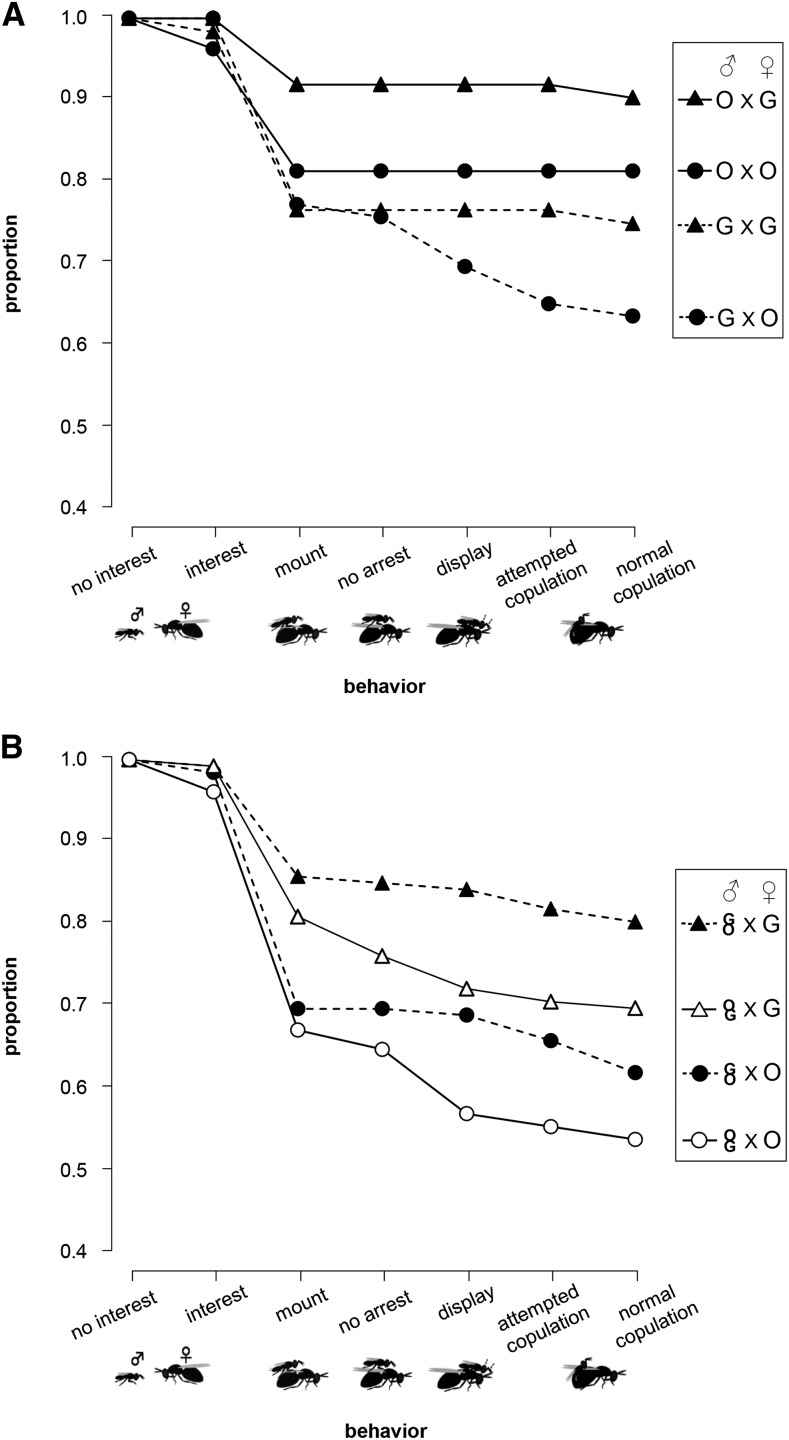
Mating behavior progress in pure species females. Proportions reaching subsequent stage in the mating behavior process are shown for (A) intraspecific and interspecific crosses of pure species, and (B) crosses of hybrid males with either parental species females. Mounting is the most discriminatory stage in both types of crosses, but subsequent stages of mating behavior in the hybrid male crosses are also more often terminated. The genotype labeling is as in Figure 1.

Several discrete stages in the *Nasonia* mating sequence are distinguished. Figure 3A shows the proportion of pairs that reach each subsequent stage for intraspecific and interspecific *N. giraulti* and *N. oneida* crosses. The majority of intraspecific crosses results in copulation, and the proportion is not very different for the two species. In interspecific crosses, *N. giraulti* females have high rates of transitions from one category to the next, resulting in overall higher proportions of completing the mating ritual than *N. oneida*, with copulation rates of 90.0% (*N. oneida* male × *N. giraulti* female) and 62.5% (*N. giraulti* male × *N. oneida* female). The main moment of interruption of the mating sequence is at the mounting stage similar to the intraspecific crosses. In contrast to the pure species crosses that are typically not interrupted after mounting, matings of *N. oneida* females, with *N. giraulti* males broke up during all subsequent stages. The same holds for matings with hybrid males which in most combinations result in lower final copulation rates than in the pure species crosses (Figure 3, A and B, see also Table S2). There is a strong female partner species effect (Table S3, ANOVA, *F*_1,490_ = 16.80, *P* < 0.001) and a grandfather species effect (Table S3, ANOVA, *F*_1,490_ = 5.64, *P* = 0.018) on copulation success in hybrid crosses. The mounting stage is again the most discriminatory (glm, effect of females: χ^2^ = 15.57, *P* < 0.001; effect of males: χ^2^ = 8.59, *P* = 0.035; no significant interaction effect; cross type effect: χ^2^ = 27.65, *P* < 0.001), with a strong female partner species effect in hybrid crosses (Table S3, ANOVA, *F*_1,490_ = 16.05, *P* < 0.001), but interruptions of the mating sequence also occur during later steps in contrast to the pure species crosses. Overall, *N. giraulti* females accept pure and hybrid males more often than do *N. oneida* females.

The number of courtship cycles of a male depends on female acceptance rate and correlates strongly with the total courtship time (correlation coefficient, *r*_559_ = 0.520, *P* < 0.001). The number of courtship cycles is consistently higher for crosses that do not result in copulation (mean ± SE; 3.48 ± 0.46 *vs.* 1.89 ± 0.06, Mann-Whitney *U*-test, *W* = 12,846, *P* = 0.049). Among intraspecific successful matings, the number of cycles is higher for *N. giraulti* than *N. oneida* (mean ± SE; 2.35 ± 0.10 *vs.* 1.02 ± 0.02, Mann-Whitney *U*-test, *W* = 1750, *P* < 0.001, Table 1). Interspecific matings with *N. oneida* females require more cycles than pure crosses (mean ± SE; 1.65 ± 0.18 *vs.* 1.02 ± 0.02, Mann-Whitney *U*-test, *W* = 1076.5, *P* = 0.001), but interspecific matings with *N. giraulti* females take fewer cycles than pure crosses (mean ± SE; 1.74 ± 0.10 *vs.* 2.35 ± 0.10, Mann-Whitney *U*-test, *W* = 634, *P* < 0.001). In hybrid crosses, a strong female partner species effect (Table S3, ANOVA, *F*_1,490_ = 10.19, *P* = 0.002) was evident on the number of courtship cycles.

**Table 1:** Total number of cycles in courtship displays that led to copulations in pure species and hybrid crosses

		Male	Female	Mean	SE	*N*	Range		Male	Female	Mean	SE	*N*	Range		*P*-Value	
Cross Type							Min	Max						Min	Max	(Mann-Whitney *U*)	
Pure species	Intra-	G	G	2.35	0.10	43	1	4	O	O	1.02	0.02	42	1	2	< 0.001	*W* = 1750
	specific																
	Inter-	G	O	1.65	0.18	40	1	5	O	G	1.74	0.10	54	1	4	0.086	*W* = 876
	specific																
Hybrid		GO	G	2.39	0.14	98	1	8	OG	G	2.18	0.15	83	0	7	0.219	*W* = 4484.5
		GO	O	1.71	0.17	76	1	7	OG	O	1.51	0.15	65	0	7	0.510	*W* = 2592.5

G, *N. giraulti*; O, *N. oneida*; GO, F_2_ male from *N. giraulti* male × *N. oneida* female cross; OG, F_2_ male from *N. oneida* male × *N. giraulti* female cross.

#### Hybrid male pheromone quantity:

**Figure 4:**
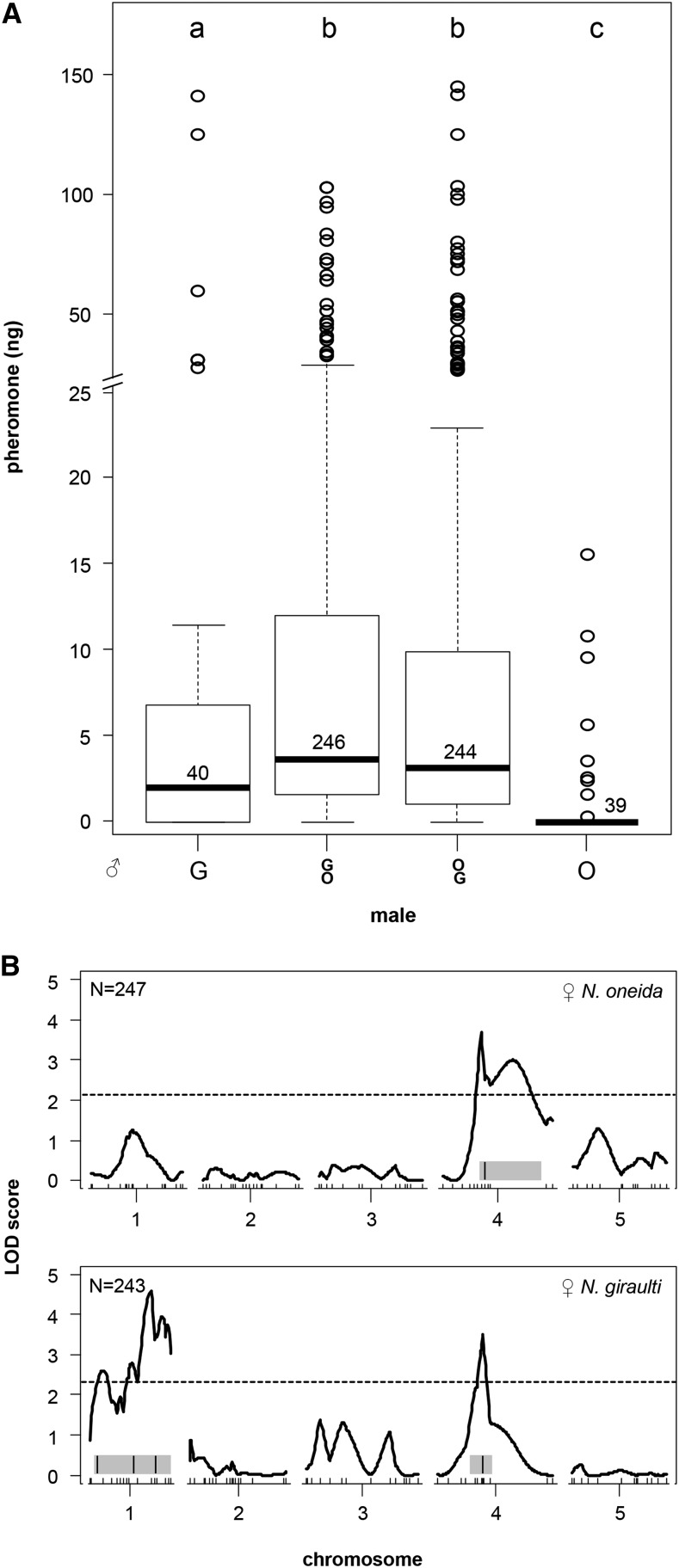
Phenotypic and QTL mapping results of pheromone quantity. (A) Phenotypic results of pheromone quantities of pure and hybrid males. *N. giraulti* males have higher pheromone quantities than *N. oneida* males. Hybrid males show many transgressive phenotypes. There is a significant interaction effect between hybrid males and female partner species. Box plots show the median (thick horizontal line within the box), the 25 and 75 percentiles (box), and 1.5 times the interquartile range of the data (thin horizontal lines). Outliers are indicated by an open circle. The genotype labeling is as in Figure 1. Sample sizes are shown within the bars. Significant differences between males are shown with letters on top of the panel. Note the *y*-axis scale break. Significant differences (Mann-Whitney *U*-test, *P* < 0.05) between crosses are shown with lower case letters. (B) QTL mapping results for male pheromone quantity. The shaded region is the 95% confidence interval for the significant QTL with the vertical line indicating the QTL peak location. The dashed line shows the 5% genome-wide significance level from permutation tests out of single-QTL genome scan. Upper and lower panel show results for males mated to *N. oneida* and *N. giraulti* females, respectively. Sample sizes are shown in the left upper corner and female partner species (backcross) in the right upper corner.

*N. giraulti* males produce the long-range sex pheromone *RS*-HDL in larger quantities than *N. oneida* males (Figure 4A, mean ± SE; 12.35 ± 4.80 ng *vs.* 1.32 ± 0.54 ng, Mann-Whitney *U*-test, *W* = 1072, *P* = 0.001). Hybrid males have on average intermediate pheromone levels with no difference between the two reciprocal crosses (mean ± SE; *giraulti–oneida* hybrid 11.00 ± 1.20 ng, *oneida–giraulti* hybrids 11.65 ± 1.47 ng, Mann-Whitney *U*-test, *W* = 32,602, *P* = 0.098), but the variation in pheromone quantities is much larger due to many transgressive phenotypes (Figure 4A). There is, however, an interaction effect between the two types of hybrid males and female partner species on pheromone quantity of hybrid males (glm, χ^2^ = 7.81, *P* = 0.005), suggesting that pheromone quantity affects female response.

**Figure 5:**
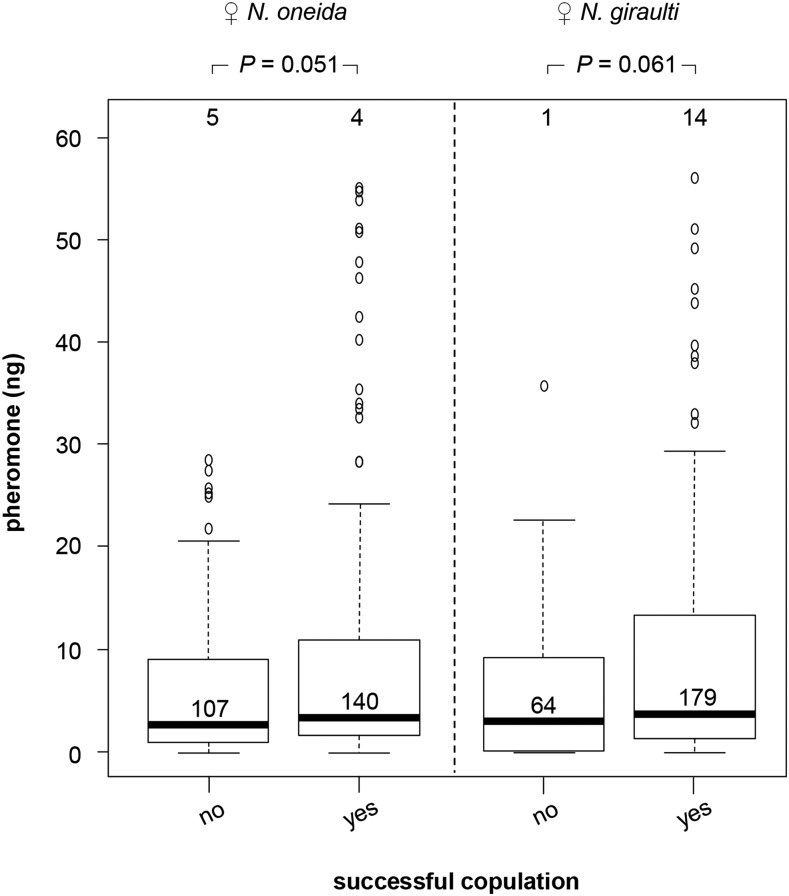
Relationship between male pheromone quantities and copulation success of hybrid males. Male pheromone quantities significantly differ between successful and unsuccessful copulations in crosses with *N. oneida* females, and almost significantly in *N. giraulti* females (Mann-Whitney *U*-test, *P* = 0.051 and *P* = 0.061, respectively). Box plots show the median (thick horizontal line within the box), the 25 and 75 percentiles (box), and 1.5 times the interquartile range of the data (thin horizontal lines). Outliers are indicated by an open circle. Pheromone quantities up to 60 ng are shown. Numbers of extra outliers higher than 60 ng are listed on top of the panel. Sample sizes are shown within the bars.

Hybrid male sex pheromone quantity has no correlation with both cross time (correlation coefficient, with *N. oneida* females, *r*_237_ = –0.095, *P* = 0.145; with *N. giraulti* females, *r*_237_ = –0.037, *P* = 0.573) and latency time (correlation coefficient, with *N. oneida* females, *r*_165_ = –0.037, *P* = 0.631; with *N. giraulti* females, *r*_199_ = –0.052, *P* = 0.458). No correlation was found between pheromone levels and number of courtship cycles (correlation coefficient, with *N. oneida* females, *r*_165_ = –0.077, *P* = 0.324; with *N. giraulti* females, *r*_199_ = 0.054, *P* = 0.443). Pheromone levels did not differ between hybrid males that successfully mounted a *N. oneida* or *N. giraulti* female compared to nonmounted males (mean ± SE; 11.64 ± 2.19 ng *vs.* 8.96 ± 1.66 ng, Mann-Whitney *U*-test, *W* = 7446, *P* = 0.145 with *N. oneida* females; and 12.75 ± 2.42 ng *vs.* 7.74 ± 1.55 ng, *W* = 4830, *P* = 0.141 with *N. giraulti* females). However, hybrid males that copulated successfully with a *N. oneida* female had almost significantly higher pheromone levels compared to unsuccessful males (Figure 5, mean ± SE; 11.49 ± 2.10 ng *vs.* 9.84 ± 1.72 ng, Mann-Whitney *U*-test, *W* = 8577, *P* = 0.051). For *N. giraulti* females this pattern was also evident and close to significance (Figure 5, mean ± SE; 13.61 ± 1.66 ng *vs.* 7.07 ± 1.72 ng, Mann-Whitney *U*-test, *W* = 6630, *P* = 0.061). This indicates that *RS*-HDL quantity affects female receptivity later in the mating process.

#### Hybrid female mate discrimination:

**Figure 6:**
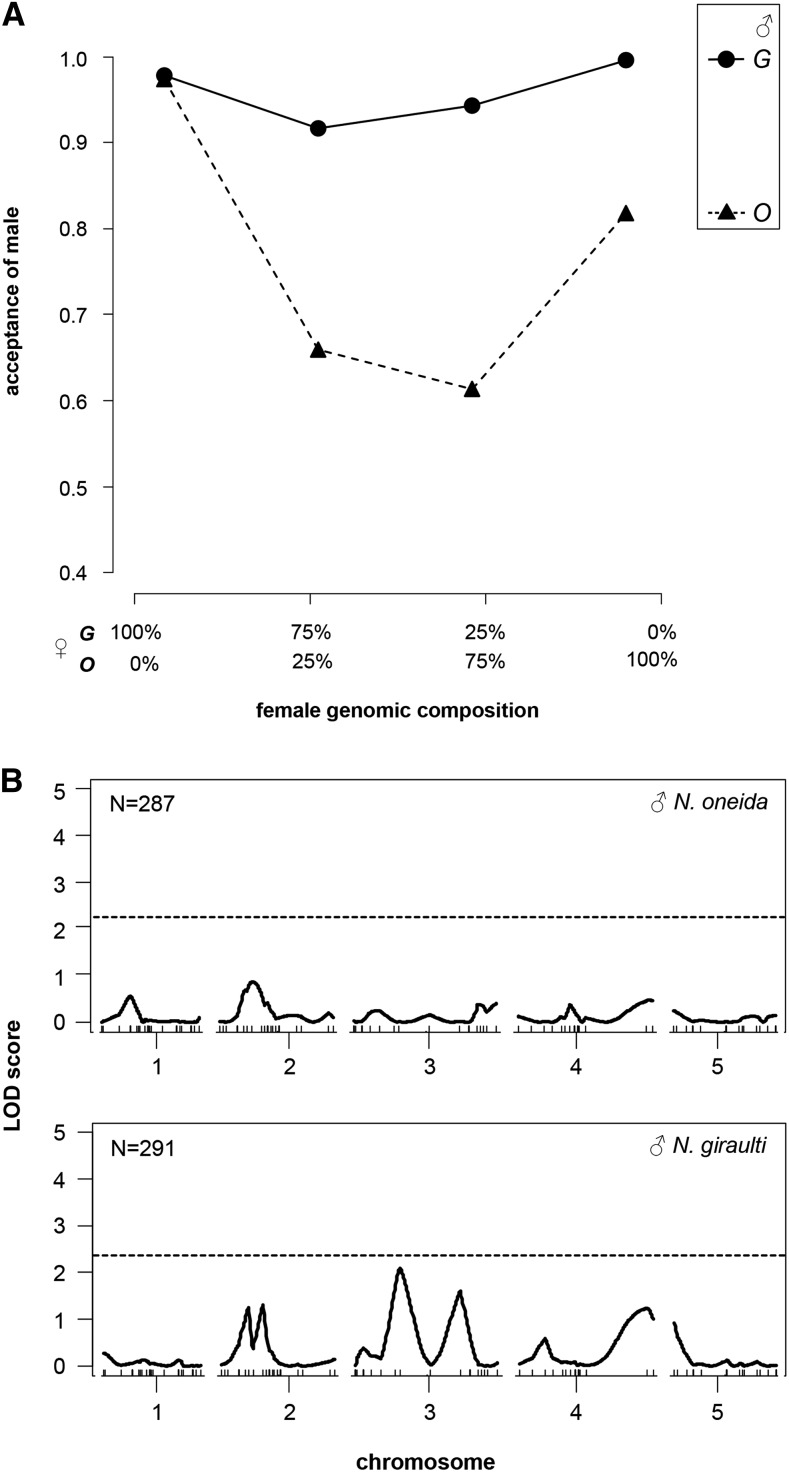
Mate discrimination. (A) Female genomic compositions with the corresponding acceptance rate of their male partner (%), and (B) QTL mapping results for female mate discrimination. Strong mate discrimination occurs in interspecific crosses between pure *N. oneida* females and pure *N. giraulti* males, and in interspecific crosses between hybrid F_3_ females whose genomic compositions were 75% of one species and 25% of the other species, and pure *N. giraulti* males but not pure *N. giraulti* females. Number of males accepted and number of pairs observed are shown in Table 2. The dashed line in (B) shows the 5% genome-wide significance level from permutation tests of the single-QTL genome scan. Upper and lower panel show results for females mated to *N. oneida* and *N. giraulti* males, respectively. Sample sizes are shown in the left upper corner, and species names of the male partner in the right upper corner.

**Figure 7:**
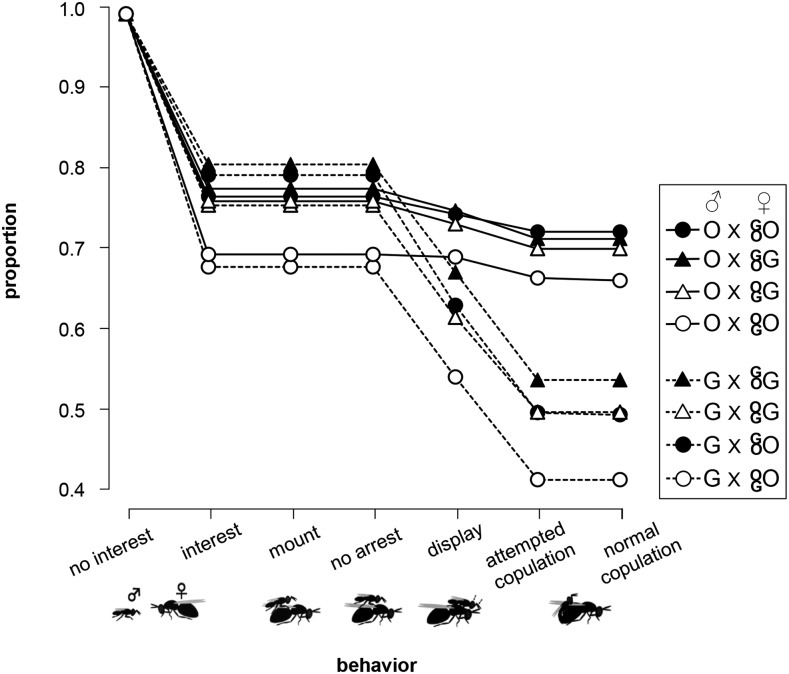
Mating behavior progress in hybrid females. Proportions reaching subsequent stage in the mating behavior process are shown for different hybrid F_3_ females. Failure of female arrest and male display occur more often in crosses with *N. giraulti* (dashed lines) than with *N. oneida* males (solid lines). The genotype labeling is as in Figure 1.

Mate discrimination is observed in interspecific crosses between pure *N. oneida* females and pure *N. giraulti* males (81.6% acceptance), as well as in interspecific crosses between hybrid F_3_ females whose genomic compositions were 75% of one species and 25% of the other species and pure *N. giraulti* males (65.3% and 60.5% acceptance) (Table 2 and Figure 6). Figure 7 shows the mating steps of hybrid F_3_ females (see also Table S2). The mating patterns differ according to male partner species (glm, effect of females: χ^2^ = 3.63, *P* = 0.304; effect of males: χ^2^ = 140.97, *P* < 0.001; no significant interaction effect; cross type effect: χ^2^ = 150.55, *P* < 0.001). Matings with *N. giraulti* males result in fewer successful copulations (708 out of 1474) than matings with *N. oneida* males (1012 out of 1448). The most prominent difference with pure species crosses (Figure 3A) is that mating disruption does not occur in the mounting step, but during the display and initial courtship (“interest”) stages. Analysis of variance for mate discrimination among F_3_ females showed a strong partner species effect (Table S3, ANOVA, *F*_1,589_ = 164.69, *P* < 0.001). Crosses with a *N. giraulti* male partner had significantly higher between sibship variances than crosses with a *N. oneida* male partner, which suggests that among-sibship genetic variation underlying female mate discrimination is expressed mainly in the presence of *N. giraulti* males but not in the presence of *N. oneida* males (Table 3 and Figure S1).

**Table 2:** Female genomic composition and their acceptance rate of the male partner

	Alleles	Percentage (%)			
Female genomic composition	*G*	100	75	25	0
	*O*	0	25	75	100
Male acceptance rate (no. male accepted / no. pairs observed)	*G*	97.7 (43/44)	65.3 (400/613)	60.5 (308/509)	81.6 (40/49)
	*O*	98.2 (54/55)	91.8 (561/611)	94.6 (451/477)	100 (42/42)

G, *N. giraulti*; O, *N. oneida*.

**Table 3:** Between- and within-sibship variance of mate discrimination in hybrid females used in the QTL analysis

Partner	Father	Mother	Offspring	Offspring	Between- Sibship Variance (*V*_p_)	Within- Sibship Variance (*V*_e_)		
			Genome	Allelic set			*P*-Value (ANOVA)	
G	GO	G	75%*G*, 25%*O*	*GG* or *GO*	26.031	0.129	< 0.001	F_1,304_ = 31.32
G	GO	O	25%*G*, 75%*O*	*GO* or *OO*	12.876	0.182	< 0.001	F_1,304_ = 70.59
G	OG	G	75%*G*, 25%*O*	*GG* or *GO*	19.38	0.16	< 0.001	F_1,284_ = 121.3
G	OG	O	25%*G*, 75%*O*	*GO* or *OO*	4.838	0.218	< 0.001	F_1,201_ = 22.18
O	GO	G	75%*G*, 25%*O*	*GG* or *GO*	1.108	0.074	< 0.001	F_1,295_ = 15
O	GO	O	25%*G*, 75%*O*	*GO* or *OO*	0.132	0.056	0.127	F_1,264_ = 2.34
O	OG	G	75%*G*, 25%*O*	*GG* or *GO*	0.297	0.066	0.034	F_1,293_ = 4.54
O	OG	O	25%*G*, 75%*O*	*GO* or *OO*	0.011	0.048	0.635	F_1,198_ = 0.24

G, *N. giraulti*; O, *N. oneida*; GO, F_2_ male from *N. giraulti* male × *N. oneida* female cross; OG, F_2_ male from *N. oneida* male × *N. giraulti* female cross.

### QTL analysis

#### Linkage map:

A linkage map corresponding to the five *Nasonia* chromosomes was constructed from the 92 SNP markers in the *N. giraulti*–*N. oneida* hybrid cross (Figure S2). None of the SNPs showed deviation from Mendelian segregation.

#### Male pheromone:

Three QTL were found at a 5% genome-wide level of significance for male pheromone quantity. One QTL was present on chromosome 4 in both the crosses with *N. giraulti* and *N. oneida* females, and this may be the same QTL as their positions overlap (Figure 4B). Another QTL was present on chromosome 1 in crosses with *N. giraulti* females only. The two-QTL scan split the QTL on chromosome 1 with *N. giraulti* partners into two QTL. The multiple-QTL scan yielded one additional QTL on chromosome 5 in crosses with *N. oneida* females. Total variances explained out of the final model are 10.2% for the QTL on chromosomes 4 and 5 in crosses with *N. oneida* females, and 14.9% for the three QTL in crosses with *N. giraulti* females (Table 4 and Figure 4B). For the QTL on chromosomes 4 and 5, hybrid males with the “*G*” allele of the associated marker (C4M8, C4M10, and C5M6) have higher pheromone levels than males with the “*O*” allele in both cross types, consistent with the larger pheromone quantities in pure *N. giraulti* males. The QTL on chromosome 1 in crosses with *N. giraulti* females had opposed effects, *i.e.*, higher pheromone quantity in crosses with *N. oneida* females (Table 4). There were significant additive effects (LOD = 7.21, *P* = 0.001), but no epistatic effects (LOD = 0.90, *P* = 0.997).

**Table 4:** Mating behavior and sex pheromone QTL

Trait	Female Crossed	Chr.	Position in cM (Range)	LOD	SNP Marker		QTL Effect (Mean ± SE)		Phenotypic Variance Explained (%)	
					Center	All	*N. giraulti*	*N. oneida*		
Cross direction	*N. oneida*	4	74 (44–108)	3.28	C4M13	C4M8*^a^*	0.66 ± 0.04	0.87 ± 0.04	6.1	
						C4M9*^a^*				
						C4M10*^a^*				
						C4M11				
						C4M12*^a^*				
						C4M13				
Copulation success	*N. giraulti*	1	4 (0–102.2)	2.67	C1M2	C1M1-C1M22 (the whole chromosome)	0.65 ± 0.04	0.84 ± 0.04	4.8	6.7
		3	17.9 (0–143.7)	1.29	C3M6	C3M1-C3M18 (the whole chromosome)	0.80 ± 0.04	0.68 ± 0.04	2.3	
Pheromone quantity	*N. oneida*	4	47 (40–108)	3.58	C4M8	C4M7	16.49 ± 1.91	5.90 ± 1.75	6.2	10.2
						C4M8*^a^*				
						C4M9*^a^*				
						C4M10*^a^*				
						C4M11				
						C4M12*^a^*				
						C4M13				
		5	28 (0–48)	2.24	C5M6	C5M1	14.53 ± 1.83	6.73 ± 1.89	3.8	
						C5M2*^a^*				
						C5M3				
						C5M4*^a^*				
						C5M5*^a^*				
						C5M6				
	*N. giraulti*	1	12 (0–28)	3.30	C1M3	C1M1*^a^*	6.64 ± 1.81	17.58 ± 1.89	5.5	14.9
						C1M2				
						C1M3				
						C1M4*^a^*				
						C1M5				
			100.1 (82.2–102.2)	2.82	C1M21	C1M17*^a^*	7.41 ± 1.84	17.46 ± 1.86	4.7	
						C1M18*^a^*				
						C1M19*^a^*				
						C1M20				
						C1M21				
						C1M22				
		4	57.7 (0–146.8)	1.27	C4M10	C4M1-C4M15 (the whole chromosome)	16.47 ± 2.00	8.29 ± 1.77	2.1	

Significant QTL with positions, effect and explained genetic variance were identified with multiple-QTL regression models.

a SNP markers corresponding to candidate genes listed in Table S1.

#### Male courtship behavior:

Because of the strong female partner species effect on male courtship, the male courtship QTL analysis was performed separately for backcross type. In the F_2_ hybrid male crosses with either pure species females, the QTL scans for cross direction yielded one QTL on chromosome 4 for crosses with *N. oneida* females, explaining 6.1% of the phenotypic variance, but none for *N. giraulti* as partners (Table 4). No significant QTL were detected at 5% significance level for border cross time, latency, mounting rate and number of cycles as part of the courtship, even after inclusion of the significant covariate partner species. If we take a 20% genome-wide significance level to further obtain suggestive QTL, peaks are visible for latency on chromosome 1 at 108 cM (LOD = 1.32, *P* = 0.122); for mounting rate on chromosome 1 at 116 cM (LOD = 0.97, *P* = 0.171); and for number of cycles, on chromosome 4 at 35.9 cM (LOD = 1.08, *P* = 0.139), and on chromosome 5 at 52 cM (LOD = 1.13, *P* = 0.126) (data not shown). There were two significant QTL at the 5% level for copulation success in crosses with *N. giraulti* females, one on chromosome 1, and one on chromosome 3, explaining 4.8% and 2.3% of the phenotypic variance, but none with *N. oneida* females. Hybrid males with the “*G*” allele of marker C1M2 have lower copulation success than males with the “*O*” allele, but the effect is opposite for marker C3M6 (Table 4).

#### Female mate discrimination:

The two reciprocal types of F_2_ hybrid males were backcrossed with either a *N. giraulti* or a *N. oneida* female, yielding a total of 2148 hybrid F_3_ females, for which mate discrimination was scored. Because female mate discrimination appeared to be expressed only in the presence of *N. giraulti* males, but not in the presence *of N. oneida* males, we performed QTL analysis separately for the two species of males (Figure 6B). As expected, we found suggestive QTL for female mate discrimination (chromosome 3, at 52.2 cM, LOD = 2.08, *P* = 0.090) when F_3_ females were mated with *N. giraulti* males (explaining 3.3% of the phenotypic variance), but no QTL when mated with *N. oneida* males. The relatively weak QTL effects detected for this trait are consistent with the fact that broad-sense heritability for this trait is only about 0.2. The inclusion of partner as both additive covariate and interactive covariate makes little difference in the QTL analysis (Figure S3).

#### Candidate genes:

Forty candidate genes for mating behavior were used as markers in our QTL analysis (Table 4 and Figure 4B). Four candidate genes on chromosome 4, *protein-1-like*, *disco*, *lateNAy*, and *Ato*, were associated with the QTL for cross direction in crosses with *N. oneida* females, and for pheromone quantity in both crosses with *N. giraulti* and *N. oneida* females. All candidate genes on chromosome 1 (*e*, *fru*, *fix_nod*, *XP001602953*, *per*, *dNA*, *cycle*, *lateNAy*, *csp*, *MpK2(2)*, and *Rhodophilin(B)*) and chromosome 3 (*TipE*, *nonA*, *dco*, *beethoven*, *headnod*, and *acyl-CoA*) were associated with QTL for copulation success in crosses with *N. giraulti* females as this QTL spans the complete chromosome. Three candidate genes on chromosome 5, *slo*, *Fmr1*, and *Dy(2)*, were associated with the QTL for pheromone quantity in crosses with *N. oneida* females. Candidate genes, *e*, *fru*, *csp*, *Mpk2(2)*, and *Rhodophilin(B)*, on chromosome 1 and an additional one, *So (1)*, on chromosome 4 were associated with QTL for pheromone quantity in crosses with *N. giraulti* females.

## Discussion

The goal of this study was to investigate the genetic architecture of male courtship traits and female preference in two recently diverged *Nasonia* species, *N. giraulti* and *N. oneida*, that exhibit strong prezygotic isolation. We performed a QTL analysis of male pheromone quantity, male courtship behavior, and female choice by crossing the two species. The differences in mate discrimination between the two species were generally confirmed in our behavioral analysis. Raychoudhury *et al.* (2010) showed that *N. oneida* females discriminate strongly against *N. giraulti* males, whereas *N. giraulti* females are less discriminatory. We also found a stronger mate discrimination of *N. oneida* females in interspecific crosses, although *N. giraulti* males were not accepted at a rate as low as 20% reported by Raychoudhury *et al.* (2010). Females were found to typically approach the males in our single pair crosses. *N. oneida* females had longer latency times than *N. giraulti* females, and these were even longer in interspecific crosses with *N. giraulti* males. There was a strong partner species effect in latency, mounting rate, number of courtship cycles, and copulation success in the hybrid male crosses. Stronger discrimination against heterospecific males was further confirmed by more frequent breaking up of mating behavior stages in interspecific crosses with *N. oneida* females, particularly in the mounting stage, resulting in lower overall copulation rate.

An important factor in the mating process is the long-range male sex pheromone component (4*R*,5*S*)-5-hydroxy-4-decanolide (*RS*-HDL), which is used to attract females from a distance (Ruther *et al.* 2007). It is produced at an almost 10-fold higher level by *N. giraulti* than *N. oneida* males. Although this pheromone functions to attract females, it apparently also affects female behavior at later stages of the mating process, such as their willingness to accept the male for copulation. We used hybrid crosses to obtain males with different pheromone quantities and tested their mating behavior in trials with pure species females. Interestingly, hybrid male pheromone quantities were sometimes higher than pure *N. giraulti* males, suggesting that pheromone production is partly disrupted (transgressive phenotypes) in hybrid individuals. Hybrid males with more pheromone quantity had significantly higher mating success with pure females of either species. Hybrid females were found to reject *N. giraulti* males more often than *N. oneida* males, but surprisingly this did not depend on their relative proportions of either species genotype. Interestingly, male rejections appeared to happen mostly during the display stage after mounting in *N. giraulti* but not in *N. oneida*, which suggest that the communication between the two partners is partly disrupted. As the long-range sex pheromone *RS*-HDL is considered to play a role before the mounting stage to attract females from a distance, a different trait may be responsible for the breaking up of the display stage. Good candidates are the cuticular hydrocarbon pattern (Buellesbach *et al.* 2013), or the short-range volatile pheromone that males produce in their mandibular glands, and deposit on the female’s antennae during male head-nods (van den Assem *et al.* 1980). Another possibility is male courtship song, which differs between species (W. Diao, L. van de Zande, M. G. Ritchie, and L. W. Beukeboom, unpublished data).

The role of the *RS*-HDL pheromone in the mating process of the various *Nasonia* species deserves further attention. *N. oneida* males apparently produce the *RS*-HDL component at much lower quantities than the other *Nasonia* species. This does not seem to negatively affect their acceptance by *N. giraulti* females, who accepted *N. oneida* males at even higher rates than their own males. In contrast, *N. oneida* females discriminate strongly against *N. giraulti* males. This suggests that the *RS*-HDL pheromone of *N. giraulti* males functions as a repellent to *N. oneida* females. A similar effect was found for the sex pheromone component (4*R*,5*R*)-5-hydroxy-4-decanolide (*RR*-HDL), which is produced only by *N. vitripennis* males, and which makes them less attractive to *N. longicornis* and *N. giraulti* females (Niehuis *et al.* 2013; Ruther *et al.* 2014). This explanation is consistent with rapid shifts in sender and receiver cues in establishing prezygotic isolation in this genus (Buellesbach *et al.* 2013; Niehuis *et al.* 2013). However, it leaves the question open as to how mate attraction occurs within the *N. oneida* species without the long-range male sex pheromone.

An important assumption in our pheromone analysis is that the amount of pheromone measured in the male abdomen after mating is representative of the amount of pheromone released before the courtship ritual to attract the female. Ruther *et al.* (2007) showed that the pheromone titer in the male abdomen depends on the age of the male. Amounts of HDL are close to zero in freshly emerged males, increase within the first 2 d after emergence, and remain at a constant level on d 3. We consistently used virgin males of 2–3 d old to control for this age effect on pheromone synthesis. It is not known how fast pheromone release depletes the amount of pheromone present in the abdominal gland when a female partner is around. As males can attract many females in a row, and a positive correlation between abdominal pheromone level and mate acceptance was found in both *Nasonia* species, our assumption of a correlation between quantity of the long-range male sex pheromone released and measured in the abdomen is likely valid.

QTL studies on hybridizing species have been informative about the underlying genetic architecture of interspecific mate discrimination. They have revealed a few QTL with large effect in some organisms though many QTL with small effects is more typically found (Coyne *et al.* 1994; True *et al.* 1997; Ritchie and Philips 1998; Doi *et al.* 2001; Takahashi *et al.* 2001; Shaw and Parsons 2002; Henry *et al.* 2002; Gleason and Ritchie 2004; Arbuthnott 2009). The vast majority of examples concern *Drosophila* species, in which male signal traits are typically components of the courtship song. Many courtship song QTL have been identified and in some cases the underlying gene has been implicated (Gleason and Ritchie 2004; Etges *et al.* 2007; Schäfer *et al.* 2010; Ellison *et al.* 2011; Lagisz *et al.* 2012; Chung *et al.* 2014). Much less is known about the genetic basis of female receptivity, but auditory and/or olfactory receptors are likely candidates (Laturney and Moehring 2012). Due to a lack of studies covering a wider array of insect species, a consistent pattern has not yet emerged about the genetic architecture of courtship differences between species. A complicating factor is that the genetic basis is often different for intraspecific and interspecific matings (*e.g.*, Gleason and Ritchie 2004; Arbuthnott 2009). Our study found two QTL for copulation success, and four for pheromone quantity on chromosomes 1, 3, 4, and 5 in the *Nasonia oneida–giraulti* species pair. Although this suggests that there may be some genomic regions with moderate effect involved in these traits, the resolution of our QTL analysis may have been limited, and QTL of minor effect may have gone undetected. Pheromone synthesis depends on many enzymatic reactions (Chung and Carroll 2015), and may therefore be considered as a complex trait. Niehuis *et al.* (2013) detected two genomic regions, one on chromosome 1 and one on chromosome 4, involved in the production of a pheromone component that is specific to *N. vitripennis*. Whether these coincide with our observed QTL on chromosomes 1 and 4 requires a higher resolution QTL study. In the study of cuticular hydrocarbon differences between *Nasonia* species, Niehuis *et al.* (2011) identified at least 42 QTL. They also listed several candidate genes for cuticular hydrocarbon production. One of these genes is *protein-1-like* (LOC100118619), which corresponds to our C4M8 marker, and that is centrally positioned under the pheromone quantity QTL on chromosome 4. As pheromone quantity was correlated with mating success, this gene is a candidate gene for mate discrimination in *Nasonia*. There are also a number of other candidate genes on chromosome 4, including *So(1)*, *disco*, *lateNAy*, and *Ato*, that were used as markers in our QTL study, and that correspond to the QTL peaks. These genes were selected for their known function in mating behavior, courtship song, circadian rhythm, or pheromone production and detection (Gleason *et al.* 2005; Kankare *et al.* 2010; Niehuis *et al.* 2013; J. Gadau, C. Pietsch, J. van den Assem, S. Gerritsma, S. Ferber, L. van de Zande, and L. W. Beukeboom, unpublished data). More detailed functional analyses, such as knock down studies, are needed to establish the possible role of these genes in *RS*-HDL quantity and interspecific mate discrimination.

Despite the large size of the mapping population (578 sibships), no significant QTL were detected for female preference at the 5% significant level, although some peaks were visible at the 20% genome-wide significance level. The broad-sense heritability of this trait is 0.2, so our results are probably consistent with a polygenic basis of female interspecific discrimination and strong environmental effects. However, the result is in contrast with two other studies. Velthuis *et al.* (2005) found a few major QTL for heterospecific male acceptance in crosses between *N. giraulti* and *N. longicornis*. We found major QTL on chromosomes 1, 2, 3, and 4 in interspecific crosses of *N. giraulti* and *N. oneida* when confronted with *N. vitripennis* males (M. C. W. G. Giesbers, B. A. Pannebakker, L. van de Zande, and L. W. Beukeboom, unpublished data). *N. giraulti* and *N. oneida* females discriminate strongly against *N. vitripennis* males, and this is mediated by the additional pheromone component, (4*R*,5*R*)-5-hydroxy-4-decanolide (*RR*-HDL) (Niehuis *et al.* 2013). Taken together, these results demonstrate that the genetic architecture for mate discrimination in the *Nasonia* species complex consists of loci with major effects, and loci with minor effects, and differs to some degree based on the species pair considered.

There are several possible explanations for the lack of female mate discrimination QTL in our study. Our experimental design did not allow for directly estimating heritability, as females were not mated with similar males. Comparison of the between-sibship variance and within-sibship variance revealed a *V*_P_ of 0.2–0.3 in crosses with *N. giraulti* male partners, consistent with values obtained from a response to selection study (M. C. W. G. Giesbers, B. A. Pannebakker, L. van de Zande, and L. W. Beukeboom, umpublished data). However, no substantial *V*_G_ was found in crosses with *N. oneida* male partners, which suggests that there is little genetic variation for mate discrimination in interspecific crosses with *N. oneida*. A possible explanation for our inability to detect mate discrimination QTL is that mate discrimination is highly polygenic and plastic, *i.e.*, with many small effect loci that went undetected because of insufficient power, and whose effect is strongly modulated by environmental conditions. Although our experimental conditions were kept as constant as possible (including temperature, humidity, time of day, and wasp age), there may have been uncontrolled factors, such as developmental differences due to host quality that affect *Nasonia* mating behavior.

Hybridization studies can be very informative about the speciation process. One major unresolved question concerns the kind of genetic changes that cause reproductive isolation. The *Nasonia* species complex is very suitable for genetic studies of speciation as different species can be intercrossed in the laboratory and confronted with heterospecific and hybrid (recombinant) mating partners. We have shown that male courtship behaviors and pheromone quantities differ between two recently diverged species and have a genetic basis. We also demonstrated that female interspecific mate discrimination in *Nasonia* is partly governed by these male traits, albeit in a complex way probably involving many genes of small effect and strong environmental effects. It is evident that the communication between the two sexes relies on multiple different cues, and that these cues partly differ in intraspecific and interspecific mating interactions. Further progress can be made with more detailed genomic studies and functional knockdowns of candidate genes in this system.

## Supplementary Material

Supplemental Material

## References

[bib1] Arbuthnott D., 2009 The genetic architecture of insect courtship behavior and premating isolation. Heredity 103: 15–22. 19259113 10.1038/hdy.2009.22

[bib2] Barton N. H. Gale K. S., 1993 Genetic analysis of hybrid zones, pp. 13–45 in *Hybrid Zones and the Evolutionary Process*, edited by R.G. Harrison Oxford University Press, New York.

[bib3] Beukeboom L. W. van den Assem J., 2001 Courtship and mating behavior of interspecific *Nasonia* hybrids (hymenoptera, pteromalidae): a grandfather effect. Behav. Genet. 31: 167–177. 11545534 10.1023/a:1010201427204

[bib4] Beukeboom L. W. van den Assem J., 2002 Courtship displays of introgressed, interspecific hybrid *Nasonia* males: further investigations into the ‘grandfather effect.’ Behaviour 139: 1029–1042.

[bib6] Bordenstein S. R. Werren J. H., 1998 Effects of a and b *Wolbachia* and host genotype on interspecies cytoplasmic incompatibility in *Nasonia*. Genetics 148: 1833–1844. 9560398 10.1093/genetics/148.4.1833 PMC1460083

[bib7] Bordenstein S. R. Drapeau M. D. Werren J. H., 2000 Intraspecific variation in sexual isolation in the jewel wasp *Nasonia*. Evolution 54: 567–573. 10937233 10.1111/j.0014-3820.2000.tb00059.x

[bib8] Bordenstein S. R. O’Hara F. P. Werren J. H., 2001 *Wolbachia*-induced incompatibility precedes other hybrid incompatibilities in *Nasonia*. Nature 409: 707–710. 11217858 10.1038/35055543

[bib9] Bordenstein S. R. Uy J. J. Werren J. H., 2003 Host genotype determines cytoplasmic incompatibility type in the haplodiploid genus *Nasonia*. Genetics 164: 223–233. 12750334 10.1093/genetics/164.1.223 PMC1462542

[bib10] Breeuwer J. A. J. Werren J. H., 1990 Microorganisms associated with chromosome destruction and reproductive isolation between two insect species. Nature 346: 558–560. 2377229 10.1038/346558a0

[bib11] Breeuwer J. A. J. Werren J. H., 1993 Effect of genotype on cytoplasmic incompatibility between two species of *Nasonia*. Heredity 70: 428–436.

[bib12] Breeuwer J. A. J. Werren J. H., 1995 Hybrid breakdown between two haplodiploid species - the role of nuclear and cytoplasmic genes. Evolution 49: 705–717. 10.1111/j.1558-5646.1995.tb02307.x 28565135

[bib13] Broman K. W. Sen S., 2009 *A Guide to QTL Mapping with R/qtl*, Springer, New York.

[bib14] Buellesbach J. Gadau J. Beukeboom L. W. Echinger F. Raychoudhury R., 2013 Cuticular hydrocarbon divergence in the jewel wasp *Nasonia*: evolutionary shifts in chemical communication channels? J. Evol. Biol. 26: 2467–2478. 24118588 10.1111/jeb.12242 PMC3809909

[bib15] Burton-Chellew M. N. Beukeboom L. W. West S. A. Shuker D. M., 2007 Laboratory evolution of polyandry in the parasitoid wasp *Nasonia vitripennis*. Anim. Behav. 74: 1147–1154.

[bib16] Chung H. Carroll S. B., 2015 Wax, sex and the origin of species: dual roles of insect cuticular hydrocarbons in adaptation and mating. BioEssays 37: 822–830. 25988392 10.1002/bies.201500014 PMC4683673

[bib17] Chung H. Loehlin D. W. Dufour H. D. Vaccarro K. Millar J. G., 2014 A single gene affects both ecological divergence and mate choice in *Drosophila*. Science 343: 1148–1151. 24526311 10.1126/science.1249998

[bib18] Clark M. E. O’Hara F. P. Chawla A. Werren J. H., 2010 Behavioral and spermatogenic hybrid male breakdown in *Nasonia*. Heredity 104: 289–301. 20087395 10.1038/hdy.2009.152 PMC2872237

[bib19] Coyne J. A., 1992 Genetics and speciation. Nature 355: 511–515. 1741030 10.1038/355511a0

[bib20] Coyne J. A. Orr H. A., 1989 Patterns of speciation in *Drosophila*. Evolution 43: 362–381. 10.1111/j.1558-5646.1989.tb04233.x 28568554

[bib21] Coyne J. A. Orr H. A., 1998 The evolutionary genetics of speciation. Philos. Trans. R. Soc. Lond. B Biol. Sci. 353: 287–305. 9533126 10.1098/rstb.1998.0210 PMC1692208

[bib22] Coyne J. A. Crittenden A. P. Man K., 1994 Genetics of a pheromonal difference contributing to reproductive isolation in *Drosophila*. Science 265: 1461–1464. 8073292 10.1126/science.8073292

[bib23] Darling D. C. Werren J. H., 1990 Biosystematics of *Nasonia* (hymenoptera, pteromalidae)—two new species reared from birds nests in North America. Ann. Entomol. Soc. Am. 83: 352–370.

[bib24] Doi M. Matsuda M. Tomaru M. Matsubayashi H. Oguma Y., 2001 A locus for female discrimination behavior causing sexual isolation in *Drosophila*. Proc. Natl. Acad. Sci. USA 98: 6714–6719. 11390998 10.1073/pnas.091421598 PMC34418

[bib25] Ellison C. K. Wiley C. Shaw K. L., 2011 The genetics of speciation: genes of small effect underlie sexual isolation in the Hawaiian cricket *Laupala*. J. Evol. Biol. 24: 1110–1119. 21375646 10.1111/j.1420-9101.2011.02244.x

[bib26] Etges W. J. de Oliveira C. C. Gragg E. Ortiz-Barrientos D. Noor M. A. F., 2007 Genetics of incipient speciation in *Drosophila mojavensis*. I. male courtship song, mating success, and genotype × environment interactions. Evolution 61: 1106–1119. 17492965 10.1111/j.1558-5646.2007.00104.x

[bib27] Gadau J. Page R. E. Werren J. H., 1999 Mapping of hybrid incompatibility loci in *Nasonia*. Genetics 153: 1731–1741. 10581280 10.1093/genetics/153.4.1731 PMC1460847

[bib28] Gadau J. Page R. E. Werren J. H. Schmid-Hempel P., 2000 Genome organization and social evolution in hymenoptera. Naturwissenschaften 87: 87–89. 10663141 10.1007/s001140050016

[bib29] Gadau J. Page R. E. Werren J. H., 2002 The genetic basis of the interspecific differences in wing size in *Nasonia* (hymenoptera; pteromalidae): major quantitative trait loci and epistasis. Genetics 161: 673–684. 12072464 10.1093/genetics/161.2.673 PMC1462138

[bib30] Gadau J. Pietsch C. Beukeboom L. W., 2012 Quantitative trait locus analysis in haplodiploid hymenoptera. Methods Mol. Biol. 871: 313–328. 22565844 10.1007/978-1-61779-785-9_16

[bib31] Giesbers M. C. W. G. Gerritsma S. Buellesbach J. Diao W. Pannebakker B. A., 2013 Prezygotic isolation in the parasitoid wasp genus *Nasonia*, pp. 165–192 in *Speciation: Natural Processes, Genetics and Biodiversity*, edited by P. Michalak Virginia Bioinformatics Institute, Virginia Tech, Blacksburg, VA.

[bib32] Gleason J. M. Ritchie M. G., 2004 Do quantitative trait loci (qtl) for a courtship song difference between *Drosophila simulans* and *D. sechellia* coincide with candidate genes and intraspecific qtl? Genetics 166: 1303–1311. 15082549 10.1534/genetics.166.3.1303 PMC1470780

[bib33] Gleason J. M. Jallon J. M. Rouault J. D. Ritchie M. G., 2005 Quantitative trait loci for cuticular hydrocarbons associated with sexual isolation between *Drosophila simulans* and *D. sechellia*. Genetics 171: 1789–1798. 16143629 10.1534/genetics.104.037937 PMC1456104

[bib34] Grant P. R. Grant B. R., 1997 Genetics and the origin of bird species. Proc. Natl. Acad. Sci. USA 94: 7768–7775. 9223262 10.1073/pnas.94.15.7768 PMC33702

[bib35] Grillenberger B. K. Koevoets T. Burton-Chellew M. N. Sykes E. M. Shuker D. M., 2008 Genetic structure of natural *Nasonia vitripennis* populations: validating assumptions of sex-ratio theory. Mol. Ecol. 17: 2854–2864. 18482258 10.1111/j.1365-294X.2008.03800.x

[bib36] Haley C. S. Knott S. A., 1992 A simple regression method for mapping quantitative trait loci in line crosses using flanking markers. Heredity 69: 315–324. 16718932 10.1038/hdy.1992.131

[bib37] Henry C. S. Wells M. L. M. Holsinger K. E., 2002 The inheritance of mating songs in two cryptic, sibling lacewing species (neuroptera: chrysopidae: chrysoperla). Genetica 116: 269–289. 12555784

[bib38] Hoffman J. I. Tucker R. Bridgett S. J. Clark M. S. Forcada J., 2012 Rates of assay success and genotyping error when single nucleotide polymorphism genotyping in non-model organisms: a case study in the Antarctic fur seal. Mol. Ecol. Resour. 12: 861–872. 22727236 10.1111/j.1755-0998.2012.03158.x

[bib39] Kankare M. Salminen T. Laiho A. Vesala L. Hoikkala A., 2010 Changes in gene expression linked with adult reproductive diapause in a northern malt fly species: a candidate gene microarray study. BMC Ecol. 10: 3–9. 20122138 10.1186/1472-6785-10-3 PMC2822739

[bib40] Koevoets T. Beukeboom L. W., 2009 Genetics of postzygotic isolation and Haldane’s rule in haplodiploids. Heredity 102: 16–23. 18523445 10.1038/hdy.2008.44

[bib41] Lagisz M. Wen S. Y. Routtu J. Klappert K. Mazzi D., 2012 Two distinct genomic regions, harbouring the period and fruitless genes, affect male courtship song in *Drosophila montana*. Heredity 108: 602–608. 22234247 10.1038/hdy.2011.129 PMC3356808

[bib42] Lander E. S. Green P., 1987 Construction of multilocus genetic-linkage maps in humans. Proc. Natl. Acad. Sci. USA 84: 2363–2367. 3470801 10.1073/pnas.84.8.2363 PMC304651

[bib43] Laturney M. Moehring A. J., 2012 Fine-scale genetic analysis of species-specific female preference in *Drosophila simulans*. J. Evol. Biol. 25: 1718–1731. 22694106 10.1111/j.1420-9101.2012.02550.x

[bib44] Lincoln S. E. Lander E. S., 1992 Systematic detection of errors in genetic linkage data. Genomics 14: 604–610. 1427888 10.1016/s0888-7543(05)80158-2

[bib45] Loehlin D. W. Enders L. S. Werren J. H., 2010 a Evolution of sex-specific wing shape at the widerwing locus in four species of *Nasonia*. Heredity 104: 260–269. 20087390 10.1038/hdy.2009.146 PMC2834783

[bib46] Loehlin D. W. Oliveira D. C. S. G. Edwards R. Giebel J. D. Clark M. E., 2010 b Non-coding changes cause sex-specific wing size differences between closely related species of *Nasonia*. PLoS Genet. 6: e1000821. 20090834 10.1371/journal.pgen.1000821 PMC2799512

[bib47] Macdonald S. J. Goldstein D. B., 1999 A quantitative genetic analysis of male sexual traits distinguishing the sibling species *Drosophila simulans* and *D. sechellia*. Genetics 153: 1683–1699. 10581276 10.1093/genetics/153.4.1683 PMC1460840

[bib48] Maniatis T. Fritsch E. F. Sambrook J., 1982 *Molecular Cloning: a Laboratory Manual*, Cold Spring Harbor Laboratory Press, New York.

[bib49] Manichaikul A. Moon J. Y. Sen S. Yandell B. S. Broman K. W., 2009 A model selection approach for the identification of quantitative trait loci in experimental crosses, allowing epistasis. Genetics 181: 1077–1086. 19104078 10.1534/genetics.108.094565 PMC2651044

[bib50] Marie Curie SPECIATION Network, 2012 What do we need to know about speciation? Trends Ecol. Evol. 27: 27–39. 21978464 10.1016/j.tree.2011.09.002

[bib51] Niehuis O. Judson A. K. Gadau J., 2008 Cytonuclear genic incompatibilities cause increased mortality in male f_2_ hybrids of *Nasonia giraulti* and *N. vitripennis*. Genetics 178: 413–426. 18202384 10.1534/genetics.107.080523 PMC2206090

[bib52] Niehuis O. Gibson J. D. Rosenberg M. S. Pannebakker B. A. Koevoets T., 2010 Recombination and its impact on the genome of the haplodiploid parasitoid wasp *Nasonia*. PLoS One 5: e8597. 20087411 10.1371/journal.pone.0008597 PMC2799529

[bib53] Niehuis O. Buellesbach J. Judson A. K. Schmitt T. Gadau J., 2011 Genetics of cuticular hydrocarbon differences between males of the parasitoid wasps *Nasonia giraulti* and *Nasonia vitripennis*. Heredity 107: 61–70. 21179062 10.1038/hdy.2010.157 PMC3186122

[bib54] Niehuis O. Buellesbach J. Gibson J. D. Pothmann D. Hanner C., 2013 Behavioral and genetic analyses of *Nasonia* shed light on the evolution of sex pheromones. Nature 494: 345–348. 23407492 10.1038/nature11838

[bib55] Nosil P., 2008 Speciation with gene flow could be common. Mol. Ecol. 17: 2103–2106. 18410295 10.1111/j.1365-294X.2008.03715.x

[bib56] Orr H. A., 2005 The genetic theory of adaptation: a brief history. Nat. Rev. Genet. 6: 119–127. 15716908 10.1038/nrg1523

[bib57] Otte D. Endler J. A., 1989 *Speciation and Its Consequences*, Sinauer Associates, Inc., Sunderland, MA.

[bib58] Presgraves D. C. Glor R. E., 2010 Evolutionary biology: speciation on islands. Curr. Biol. 20: R440–R442. 20504751 10.1016/j.cub.2010.03.032

[bib59] Presgraves D. C. Balagopalan L. Abmayr S. M. Orr H. A., 2003 Adaptive evolution drives divergence of a hybrid inviability gene between two species of *Drosophila*. Nature 423: 715–719. 12802326 10.1038/nature01679

[bib60] Pultz M. A. Leaf D. S., 2003 The jewel wasp *Nasonia*: querying the genome with haplo-diploid genetics. Genesis 35: 185–191. 12640624 10.1002/gene.10189

[bib61] Raychoudhury R. Desjardins C. A. Buellesbach J. Loehlin D. W. Grillenberger B. K., 2010 Behavioral and genetic characteristics of a new species of *Nasonia*. Heredity 104: 278–288. 20087394 10.1038/hdy.2009.147 PMC3533498

[bib62] Rice W. R. Hostert E. E., 1993 Laboratory experiments on speciation—what have we learned in 40 years. Evolution 47: 1637–1653. 10.1111/j.1558-5646.1993.tb01257.x 28568007

[bib63] Ritchie M. G. Phillips S. D. F., 1998 The genetics of sexual isolation, pp. 291–308 in *Endless Forms: Species and Speciation*, edited by D. J. Howard Berlocher S. H. Oxford University Press, New York.

[bib64] Ruther J. Stahl L. M. Steiner S. Garbe L. A. Tolasch T., 2007 A male sex pheromone in a parasitic wasp and control of the behavioral response by the female’s mating status. J. Exp. Biol. 210: 2163–2169. 17562890 10.1242/jeb.02789

[bib65] Ruther J. Steiner S. Garbe L. A., 2008 4-methylquinazoline is a minor component of the male sex pheromone in *Nasonia vitripennis*. J. Chem. Ecol. 34: 99–102. 18085340 10.1007/s10886-007-9411-1

[bib66] Ruther J. Thal K. Blaul B. Steiner S., 2010 Behavioral switch in the sex pheromone response of *Nasonia vitripennis* females is linked to receptivity signalling. Anim. Behav. 80: 1035–1040.

[bib67] Ruther J. McCaw J. Boecher L. Pothmann D. Putz I., 2014 Pheromone diversification and age-dependent behavioral plasticity decrease interspecific mating costs in *Nasonia*. PLoS One 9: e89214. 24551238 10.1371/journal.pone.0089214 PMC3925242

[bib68] Schäfer M. A. Mazzi D. Klappert K. Kauranen H. Vieira J., 2010 A microsatellite linkage map for *Drosophila montana* shows large variation in recombination rates, and a courtship song trait maps to an area of low recombination. J. Evol. Biol. 23: 518–527. 20040000 10.1111/j.1420-9101.2009.01916.x

[bib69] Sharon G. Segal D. Zilber-Rosenberg I. Rosenberg E., 2011 Symbiotic bacteria are responsible for diet-induced mating preference in *Drosophila melanogaster*, providing support for the hologenome concept of evolution. Gut Microbes 2: 190–192. 21804354 10.4161/gmic.2.3.16103

[bib70] Shaw K. L. Parsons Y. M., 2002 Divergence of mate recognition behavior and its consequences for genetic architectures of speciation. Am. Nat. 159: 61–75. 10.1086/338373 18707370

[bib71] Shaw K. L. Parsons Y. M. Lesnick S. C., 2007 Qtl analysis of a rapidly evolving speciation phenotype in the Hawaiian cricket *Laupala*. Mol. Ecol. 16: 2879–2892. 17614904 10.1111/j.1365-294X.2007.03321.x

[bib72] Steiner S. Ruther J., 2009 Mechanism and behavioral context of male sex pheromone release in *Nasonia vitripennis*. J. Chem. Ecol. 35: 416–421. 19390897 10.1007/s10886-009-9624-6

[bib73] Steiner S. Hermann N. Ruther J., 2006 Characterization of a female-produced courtship pheromone in the parasitoid *Nasonia vitripennis*. J. Chem. Ecol. 32: 1687–1702. 16900425 10.1007/s10886-006-9102-3

[bib74] Takahashi A. Tsaur S. C. Coyne J. A. Wu C. I., 2001 The nucleotide changes governing cuticular hydrocarbon variation and their evolution in *Drosophila melanogaster*. Proc. Natl. Acad. Sci. USA 98: 3920–3925. 11259658 10.1073/pnas.061465098 PMC31154

[bib75] Tamura K. Peterson D. Peterson N. Stecher G. Nei M., 2011 Mega5: molecular evolutionary genetics analysis using maximum likelihood, evolutionary distance, and maximum parsimony methods. Mol. Biol. Evol. 28: 2731–2739. 21546353 10.1093/molbev/msr121 PMC3203626

[bib76] True J. R. Liu J. J. Stam L. F. Zeng Z. B. Laurie C. C., 1997 Quantitative genetic analysis of divergence in male secondary sexual traits between *Drosophila simulans* and *Drosophila mauritiana*. Evolution 51: 816–832. 10.1111/j.1558-5646.1997.tb03664.x 28568599

[bib77] van den Assem J., 1986 Mating behavior in parasitic wasps, pp. 137–167 in Insect Parasitoids; 13th *Symposium of the Royal Entomological Society of London*, edited by J. K. Waage Greathead D. J., Academic Press, London.

[bib78] van den Assem J. Werren J. H., 1994 A comparison of the courtship and mating behavior of three species of *Nasonia* (hymenoptera, pteromalidae). J. Insect Behav. 7: 53–66.

[bib79] van den Assem J. Beukeboom L. W., 2004 A review of *Nasonia* (chalcidea, pteromalidae) courtship and mating behavior, with some additional new observations. Proc. Neth. Entomol. Soc. 15: 123–132.

[bib80] van den Assem J. Jachmann F. Simbolotti P., 1980 Courtship behavior of *Nasonia vitripennis* (hym., pteromalidae): some qualitative, experimental evidence for the role of pheromones. Behaviour 75: 301–307.

[bib81] Velthuis B. J. Yang W. C. van Opijnen T. Werren J. H., 2005 Genetics of female mate discrimination of heterospecific males in *Nasonia* (hymenoptera, pteromalidae). Anim. Behav. 69: 1107–1120.

[bib82] Walker F., 1836 Monographia chalciditum. Entomological Magazine 3: 465–496.

[bib83] Werren J. H. Loehlin D. W., 2009 The parasitoid wasp *Nasonia:* an emerging model system with haploid male genetics. Cold Spring Harbor Protoc. 10: pdb.emo134. 10.1101/pdb.emo134 PMC2916733 20147035

[bib84] Werren J. H. Richards S. Desjardins C. A. Niehuis O. Gadau J., 2010 Functional and evolutionary insights from the genomes of three parasitoid *Nasonia* species. Science 327: 343–348. 20075255 10.1126/science.1178028 PMC2849982

[bib85] Whiting A. R., 1967 The biology of parasitic wasp *Mormoniella vitripennis* (=*Nasonia brevicornis*) (walker). Q. Rev. Biol. 42: 333–406.

