# Supplementary material for: Quantitative Trait Locus Analysis of Mating Behavior and Male Sex Pheromones in Nasonia Wasps
Source: G3 (Bethesda). 2016 Mar 26;6(6):1549–62. doi: 10.1534/g3.116.029074 (PMC4889652; doi:10.1534/g3.116.029074)
Supplement: Supplemental Material [file supp_6_6_1549__index.html]

Supplemental Material 

# Quantitative Trait Locus Analysis of Mating Behavior and Male Sex Pheromones in *Nasonia* Wasps

Supplemental Material for Diao *et al.*, 2016

Supplemental Material

**Files in this Data Supplement:**

- Figure S1 - Mate discrimination of hybrid females used for QTL mapping. (.pdf, 32 KB)
- Figure S2 - Linkage map of the *N. giraulti-N. oneida* hybrid crosses with 92 SNP markers. (.pdf, 46 KB)
- Figure S3 - QTL mapping results included partner as a covariate and QTL x partner as an interaction covariate for female mate discrimination. (.pdf, 23 KB)
- Table S1 - Details of SNP markers. (.xlsx, 172 KB)
- Table S2 - Number of *Nasonia* pairs for each distinguished mating behavior stage as depicted in Figure 3 and 7. (.pdf, 19 KB)
- Table S3 - Covariate effects on all mating behavior traits in hybrid crosses. (.pdf, 117 KB)
- Table S4 - Courtship behavior data. (.xlsx, 80 KB)
- Table S5 - Male pheremone quantity data. (.xlsx, 34 KB)
- Table S6 - Female mate discrimination data. (.xlsx, 226 KB)
- Table S7 - F2 hybrid male genotypic data. (.xlsx, 169 KB)
