# Supplementary material for: Quantitative Trait Locus Analysis of Mating Behavior and Male Sex Pheromones in Nasonia Wasps
Source: G3 (Bethesda). 2016 Mar 26;6(6):1549–62. doi: 10.1534/g3.116.029074 (PMC4889652; doi:10.1534/g3.116.029074)
Supplement: Supplemental Material [file supp_g3.116.029074_FigureS1.pdf]

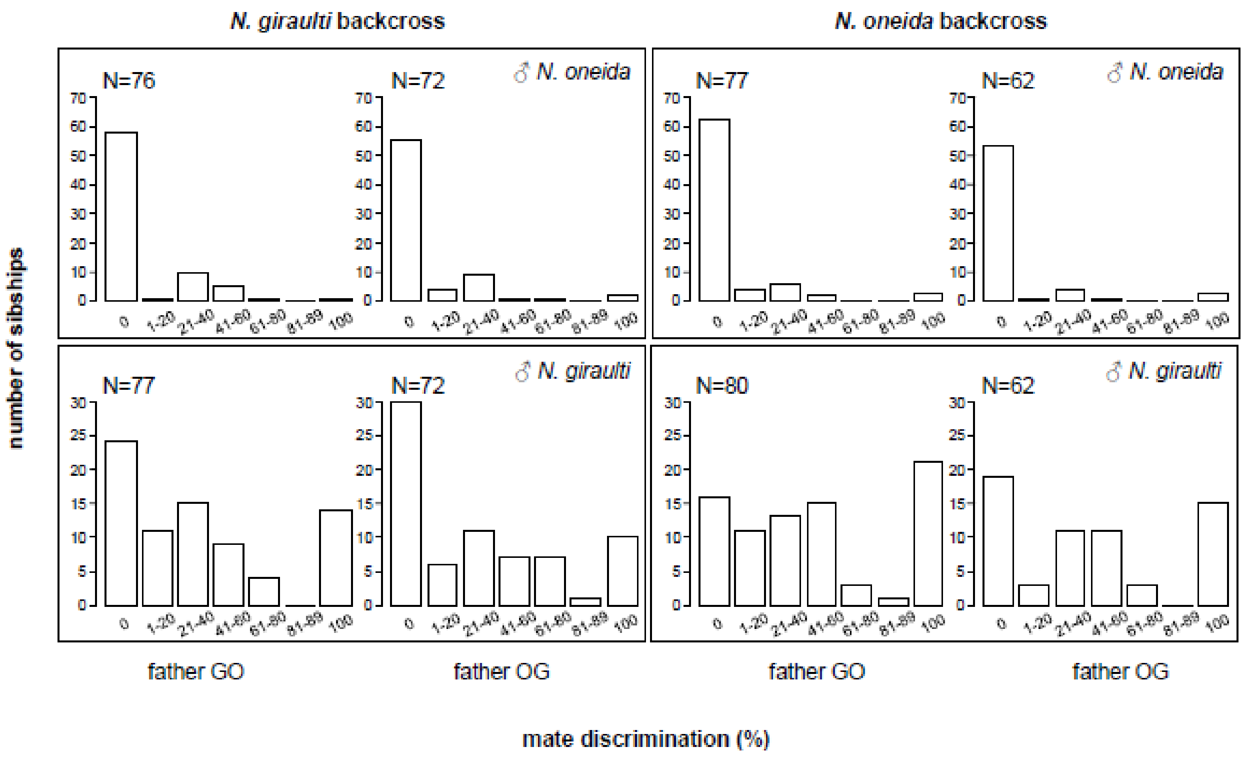

Figure S1 Mate discrimination of hybrid females used for QTL mapping. Paternal genotypes are shown below the four panels, and species names of the partners in the upper right corner of each panel.
