# Supplementary material for: Quantitative Trait Locus Analysis of Mating Behavior and Male Sex Pheromones in Nasonia Wasps
Source: G3 (Bethesda). 2016 Mar 26;6(6):1549–62. doi: 10.1534/g3.116.029074 (PMC4889652; doi:10.1534/g3.116.029074)
Supplement: Supplemental Material [file supp_g3.116.029074_FigureS2.pdf]

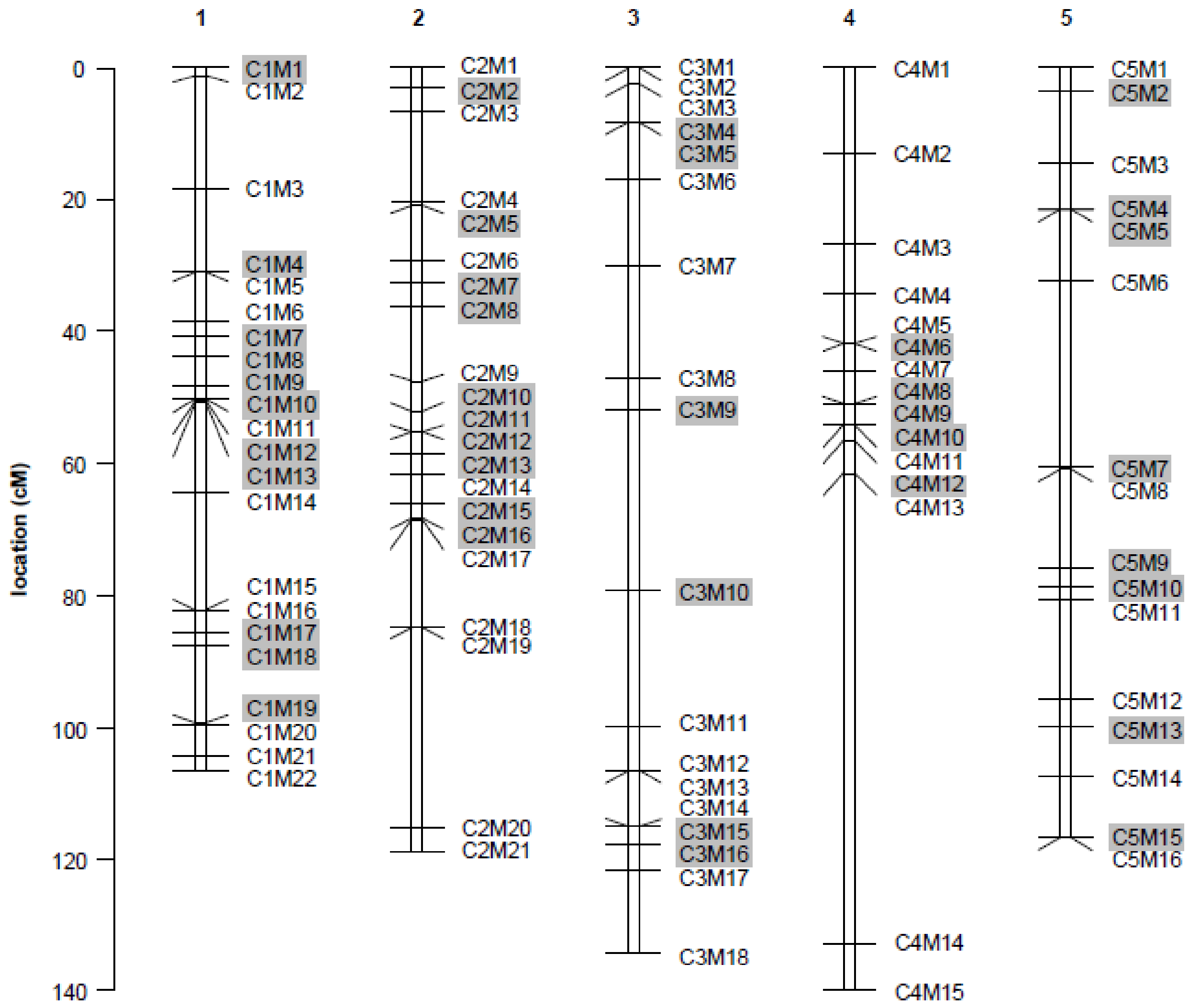

Figure S2 Linkage map of the *N. giraulti-N. oneida* hybrid crosses with 92 SNP markers. The five linkage groups correspond to the five *Nasonia* chromosomes. The y-axis shows recombination distance in centiMorgans. SNP markers with grey shading correspond to candidate genes listed in Table S1.
