# Supplementary material for: Quantitative Trait Locus Analysis of Mating Behavior and Male Sex Pheromones in Nasonia Wasps
Source: G3 (Bethesda). 2016 Mar 26;6(6):1549–62. doi: 10.1534/g3.116.029074 (PMC4889652; doi:10.1534/g3.116.029074)
Supplement: Supplemental Material [file supp_g3.116.029074_FigureS3.pdf]

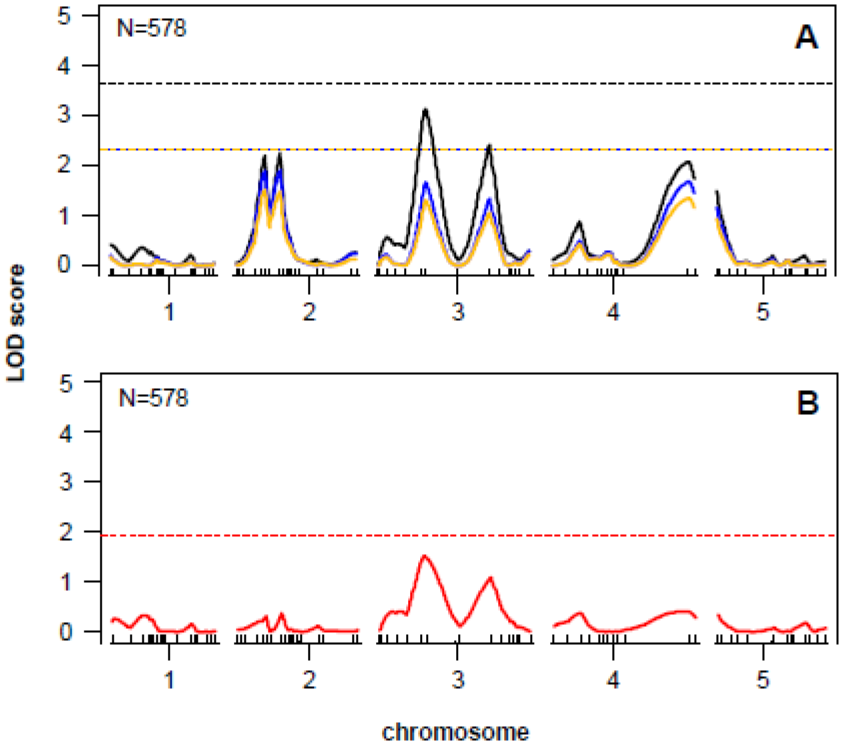

Figure S3 QTL mapping results included partner as a covariate and QTL x partner as an interaction covariate for female mate discrimination. Panel A shows the QTL results without covariate (yellow), with additive covariate (blue), and with both additive and interactive covariate (black). Panel B shows the QTL results with interactive covariate only. The dashed line shows the 5% genome-wide significance level from permutation tests of the single-QTL genome scan. The dashed lines in Panel A with color yellow and blue are overlapped.
