# Supplementary material for: Quantitative Trait Locus Analysis of Mating Behavior and Male Sex Pheromones in Nasonia Wasps
Source: G3 (Bethesda). 2016 Mar 26;6(6):1549–62. doi: 10.1534/g3.116.029074 (PMC4889652; doi:10.1534/g3.116.029074)
Supplement: Supplemental Material [file supp_g3.116.029074_TableS2.pdf]

Table S2 Number of *Nasonia* pairs for each distinguished mating behavior stage as depicted in Figure 3 and 7

	male	female	mating behavior stages						
			no interest	interest	mount	no arrest	display	attempted copulation	normal copulation
Figure 3A	G	G	58	57	44	44	44	44	43
	G	O	64	64	49	48	44	41	40
	O	G	60	60	55	55	55	55	54
	O	O	52	50	42	42	42	42	42
Figure 3B	GO	G	123	122	105	104	103	100	98
	GO	O	125	123	86	86	85	81	76
	OG	G	122	121	98	82	87	85	84
	OG	O	124	119	82	79	69	67	65
Figure 7	G	GO-G	383	309	309	309	256	203	203
	G	GO-O	377	299	299	299	236	184	183
	G	OG-G	403	304	304	304	246	197	197
	G	OG-O	311	210	210	210	166	125	125
	O	GO-G	388	301	301	301	290	276	276
	O	GO-O	354	271	271	271	263	255	255
	O	OG-G	408	310	310	310	298	285	285
	O	OG-O	298	206	206	206	205	197	196

G = *N. giraulti*; O = *N. oneida*; GO = F_2 male from *N. giraulti* male x *N. oneida* female cross; OG = F_2 male from *N. oneida* male x *N. giraulti* female cross; GO-G = F_3 female from GO male x *N. giraulti* female cross; GO-O = F_3 female from GO male x *N. oneida* female cross; OG-G = F_3 female from OG male x *N. giraulti* female cross; OG-O = F_3 female from OG male x *N. oneida* female cross.
