# Supplementary material for: Quantitative Trait Locus Analysis of Mating Behavior and Male Sex Pheromones in Nasonia Wasps
Source: G3 (Bethesda). 2016 Mar 26;6(6):1549–62. doi: 10.1534/g3.116.029074 (PMC4889652; doi:10.1534/g3.116.029074)
Supplement: Supplemental Material [file supp_g3.116.029074_TableS3.pdf]

Table S3 Covariate effects on all mating behavior traits in hybrid crosses

covariate	hybrid males traits							hybrid female traits
	cross direction	cross time	latency time	mounting	number of cycles	copulation success	male pheromone	female mate discrimination
partner	0.427	0.996	<0.001*	<0.001*	0.001*	<0.001*	0.545	<0.001*
grandmother	√	√	√	√	√	√	√	
grandfather	0.465	0.820	0.888	0.317	0.250	0.178	0.732	
partner:grandmother	√	√	√	√	√	√	√	
partner:grandfather	0.608	0.373	0.888	0.758	0.299	0.699	0.006*	
partner:grandmother:grandfather	√	√	√	√	√	√	√	
mother								0.097
father								0.527
partner:mother								0.016*
partner:father								0.527
mother:father								0.947
partner:mother:father								0.882

Blank cells indicate no information available. √ tested, but not detected.

Analysis of variance (ANOVA) was used to estimate covariate effects on all measured mating behavior traits in hybrid crosses. Effect of partner, grandfather and grandmother genotype and their interaction were estimated for hybrid male traits. Effect of partner, father and mother genotype and their interaction were estimated for hybrid female traits. The factors with significant effects on the traits are marked with “*” (*P* < 0.05).
